# Anthraquinones and Derivatives from Marine-Derived Fungi: Structural Diversity and Selected Biological Activities

**DOI:** 10.3390/md14040064

**Published:** 2016-03-25

**Authors:** Mireille Fouillaud, Mekala Venkatachalam, Emmanuelle Girard-Valenciennes, Yanis Caro, Laurent Dufossé

**Affiliations:** 1 Laboratoire de Chimie des Substances Naturelles et des Sciences des Aliments—LCSNSA EA 2212, Université de la Réunion, 15 Avenue René Cassin, CS 92003, F-97744 Saint-Denis Cedex 9, Ile de la Réunion, France; mekalavenkat@gmail.com (M.V.); emmanuelle.girard-valenciennes@univ-reunion.fr (E.G.-V.); yanis.caro@univ-reunion.fr (Y.C.); laurent.dufosse@univ-reunion.fr (L.D.); 2 Ecole Supérieure d′Ingénieurs Réunion Océan Indien—ESIROI, 2 Rue Joseph Wetzell, F-97490 Sainte-Clotilde, Ile de la Réunion, France

**Keywords:** anthraquinone, marine, fungi, pigment, biological activity, antitumor, antibiotic, cytotoxicity

## Abstract

Anthraquinones and their derivatives constitute a large group of quinoid compounds with about 700 molecules described. They are widespread in fungi and their chemical diversity and biological activities recently attracted attention of industries in such fields as pharmaceuticals, clothes dyeing, and food colorants. Their positive and/or negative effect(s) due to the 9,10-anthracenedione structure and its substituents are still not clearly understood and their potential roles or effects on human health are today strongly discussed among scientists. As marine microorganisms recently appeared as producers of an astonishing variety of structurally unique secondary metabolites, they may represent a promising resource for identifying new candidates for therapeutic drugs or daily additives. Within this review, we investigate the present knowledge about the anthraquinones and derivatives listed to date from marine-derived filamentous fungi′s productions. This overview highlights the molecules which have been identified in microorganisms for the first time. The structures and colors of the anthraquinoid compounds come along with the known roles of some molecules in the life of the organisms. Some specific biological activities are also described. This may help to open doors towards innovative natural substances.

## 1. Introduction

In recent decades, marine organisms focused the attention of researchers for their huge potential in producing bioactive compounds [1,2,3,4]. Among them, the microorganisms gradually took on an important role because they appear to be prolific producers of a wide diversity of secondary metabolites [5,6]. Cultivated in bioreactors, they also represent a sustainable and easily “upscalable” resource, therefore not endangering fragile marine ecosystems. Among these microorganisms, fungi, which had their terrestrial glory days after the discovery of penicillin, are again at the forefront in the search for new marine molecules in view of their rich biodiversity, even in deep-sea niches [7]. Around 70,000 fungal species have already been described worldwide, and among them about 1500 species of marine-derived fungi were mentioned, primarily from coastal ecosystems [8,9]. As 70% of the earth is submerged, Gareth Jones (1998) [10] estimated the total number of marine and marine-derived fungal species to be a minimum of 72,000, indicating that the inherent discovery of new compounds is still in its infancy. Several fungal metabolites from marine origin have already demonstrated their originality and efficacy in different domains. As an example, in the field of therapeuthics, we can mention the two chemically unusual cyclodepsipeptides patented in 2008—scopularides A and B—from the marine-derived *Scopulariopsis brevicaulis*, demonstrating anticancer activities [11,12], as well as the halimide (plinabuline) from an endophytic *Aspergillus* sp. *CNC-139* isolated from the green algae *Halimeda lacrymosa*, already achieving phase II clinical tests [13].

Biosynthetically, many extrolites produced by filamentous fungi are polyketides, and several papers report that polyketides seem to dominate marine natural products of fungal origin [14,15]. Polyketides represent an array of often structurally complex natural products including such classes as anthraquinones, hydroxyanthraquinones, naphthalenes, naphthoquinones, flavonoids, macrolides, polyenes, tetracyclines, and tropolones. Many of them have already exhibited either positive or negative effects, as wide as those that are antimicrobial, anticancer, antioxidant, immunomodulatory, cytotoxic, or carcinogenic. This closely concerns the class of anthraquinones whose effects, depending on the nature and amount of compound, can either be beneficial or noxious towards living organisms. These compounds, little studied because of their bad reputation, mainly arising from their benzenic patterns, are, however, worthy of the same attention as other families of fungal compounds, whose members have become pillars of the global pharmacopeia (antibiotics) and are widely used in food or staining industries (azaphilone colorants from *Monascus* spp. in Asia). Within this review, we investigate the present knowledge of the anthraquinonoid compounds listed to date from marine-derived filamentous fungi′s productions. This overview highlights the molecules identified for the first time and comes along with interesting characteristics: the panel of colors, their known roles in the biology of the organisms, and some specific *in vitro* biological activities. As the natural products constitute the dynamic element of the present global market, we hope this review can help broadening the horizon towards innovative substances.

## 2. Anthraquinones from Marine-Derived Fungi

About 700 anthraquinone derivatives were identified in plants, lichens, and fungi; 43 have already been described from fungal cultures [16,17]. Due to their structure, they exhibit interesting chromatic properties and decline a wide range of nuances in colors. Thus, they first presented a great interest in the field of dyeing molecules, highly requested in cosmetics, clothes dyeing and foodstuff industries. From their structures, hydroxyanthraquinone pigments have a relative stability. They also possess good light-fastness properties, which often makes metallization unnecessary. Nevertheless, they can easily form complexes with several metal salts (or cations in general) (aluminium, barium, calcium, copper, palladium, iron) [18,19,20,21,22] and exhibit superior brightness compared to azo-pigments [23,24]. This capacity to form metallic complexes is of a great interest in an industrial context: The complex forms often reduce the solublity in water, enhancing the solvent solubility, without loosing the brightness [20]. In the textile industry, hydroxyanthraquinone are, moreover, considered “reactive dyes,” as they form a covalent bond with the fibers, usually cotton, although they are used to a small extent on wool and nylon. Therefore, they have it it possible to achieve extremely high wash fastness properties by relatively simple dyeing methods. Thereby, the literature abundantly reports the interest for marine organisms with respect to the production of new molecules and, among them, new pigments [25,26]. Besides their coloring properties, anthraquinoid compounds exhibit a wide range of diverse biological activities, sparsely mentioned in the literature. Regarding this aspect, their “Dr Jekyll and Mr Hyde” physiognomy [27] needs to be carefully elucidated in order to examine their potential use in pharmaceutical or alimentary fields, with maximum objectivity.

### 2.1. Anthraquinone′s Basic Structure

**Figure 1:**
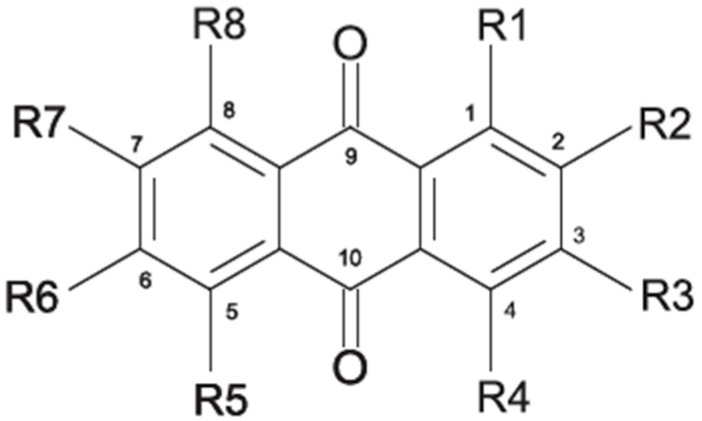
Anthraquinone general structure (R1–R8: lateral substituents).

Anthraquinones represent a class of molecules of the quinone family, based on a structure composed of three benzene rings. The basic structure 9,10-anthracenedione, also called 9,10-dioxoanthracene (formula C_14_H_8_O_2_), includes two ketone groups on the central ring (Figure 1). The diversity of the anthraquinoid compounds relies on the nature and the position of the substituents, replacing the H atoms on the basic structure (R1 to R8), as diverse as: –OH, –CH_3_, –OCH_3_, –CH_2_OH, –CHO, –COOH, or more complex groups. When *n* hydrogen atoms are replaced by hydroxyl groups, the molecule is called hydroxyanthraquinone (HAQN). From their structure, HAQN derivatives absorb visible light and are colored.

An important characteristic of the anthraquinone compound is their electronic absorption spectra. The strong absorption in the ultraviolet region is due to the presence of chromophore formed by the system of conjugated double bonds. The spectra of anthraquinone are highly complex because of the presence of absorption bands due to the benzenoid transitions, in addition to quinonoid absorptions. The benzenoid bands appear fairly regularly within the range 240–260, with intense absorption at 250 nm and in 320–330 nm, and with medium absorption at 322 nm, whereas the quinonoid bands absorb at 260–290 nm. These areas of selective absorption are characteristic, and the pattern in the ultraviolet region is not seriously affected by substitution. In addition, hydroxyl anthraquinones show an absorption band(s) at 220–240 nm, not shown by the parent compound. In the visible area, an unsubstituted anthraquinone has a weak yellow color, and its electronic absorption spectrum contains a small peak at 405 nm. The presence of substituents in position 1 and 4 induces a significant bathochromic shift, intensifying the color more significantly than the substituents in the 1,5 and 1,8 positions. Thus, with an alcoholic solution of magnesium acetate, 1,2-dioxyderivative is colored in violet; 1,4-dioxyderivative in purple; and 1,8-dioxyderivative in red-orange [28,29,30]. Therefore, fungal anthraquinones range from pale yellow to dark red or brown colors, through to violet.

### 2.2. Ecology of Marine-Derived Fungal Anthraquinones Producers

**Figure 2:**
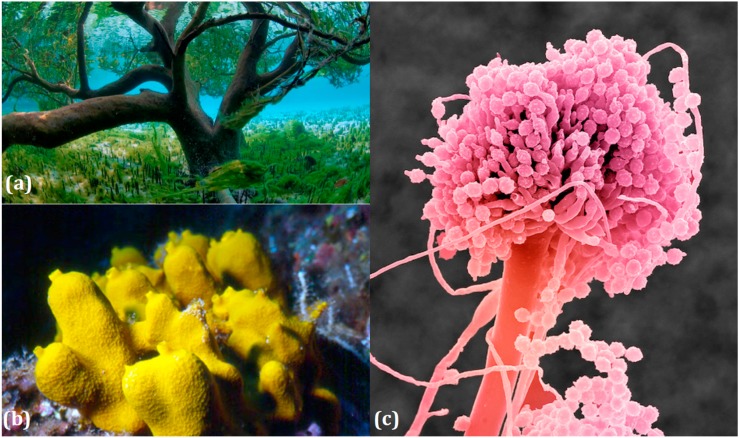
Marine habitats hosting fungal anthraquinones producers. (**a**) Tree and marine plants growing in a submerged area (**b**) *Aplysina aerophoba* (mediterranean sponge), usual host of endophytic filamentous fungi; (**c**) *Aspergillus versicolor*, exhibiting a pink pigment.

Marine ecosystems host a wide biodiversity of filamentous fungi found in free waters, inert organic or inorganic matter. They can also be included as endophytes or pathogens in marine plants, planktons, vertebrates and invertebrates [31]. Their different roles in these environments are still poorly known, although their implications in lignocellulolytic compounds degradation and mineralization of organic matter has been repeatedly demonstrated [7,32,33]. Yet the notion of “marine fungus” is still under debate in the world of mycologists. Fungi are usually recognized as ubiquitous because they inhabit a plethora of ecosystems, from terrestrial milieus to aquatic environments (Figure 2). Marine and marine-derived fungi therefore form an ecological, not a taxonomic, group [34]. From the widely adopted definition of Kohlmeyer *et al.* (1979) [8], they are divided into two ecotypes:

- obligate marine fungi (true ones) that grow and sporulate only in seawater. Their spores are able to germinate and form new thalli in salted environment.
- transitional marine fungi (marine-derived fungi) that come from terrestrial or freshwater media and have undergone physiological adaptation to survive, grow, or reproduce in the marine environment.

In fungi, anthraquinones are produced from different steps or branches of the polyketides pathway. Today, it is clear that, as far as secondary metabolites and *a priori* anthraquinoid productions are concerned, a great variability appears among species of the same genus, even among strains in the same species. This could undoubtly be related to the capacities a fungus has to develop, in order to face some specific conditions in specific ecosystems. As an illustration, the composition of the quinoid pigment complexes of *P. funiculosum* strains isolated from various types of soils are quite different when cultivated in the same artificial culture media [35]. That is why, even if the polyketides pathway is mentioned in a strain, not all strains inside the species are anthraquinones producers. In the same way, if the fungal metabolism is able to express anthtraquinones and (simultaneously) to excrete toxins, the presence of these secondary products are highly dependent on external physico-chemical conditions [36].

Thus, a high diversity of molecules is now expected from unexplored marine-derived fungi, which are considered promising novel sources of chemical diversity. The potential of marine-derived microorganisms to produce unique and original molecules could therefore come from specific metabolic or genetic adaptations appearing to meet very specific combinations of physico-chemical parameters (high osmotic pressure, low O_2_ penetration, low temperature, limited light access, high pressure, or regular tidal ebbs and flows) [37]. Indeed, the two marine ecotypes lead to particular behaviors and consecutively to specific products, compared to the terrestrial congeners: either the challenge of facing unusual living conditions (exogenous fungi) or the use of specific procedures naturally adapted to the marine niches (*i.e*., indigenous micromycetes, naturally selected for aquatic environments). This skill is, for instance, exemplified by marine macroorganisms′ fungal endophytes as corals or sponges. For now, the highest diversity of marine-derived fungi seems to be found in tropical regions, mainly in tropical mangroves, which are extensively studied because of their high richness in organic matters. Obviously, these biotopes seem favorable to the development of a high diversity of heterotrophic microorganisms based on the diversity of organic and inorganic substrates [8,38].

The questions on the effect of interactions between organisms on microbes extrolites is amply fueled in the case of a very producive lichen′s symbioses. A lichen is a composite organism that emerges from algae or cyanobacteria (or both) living with filaments of a fungus in a mutually beneficial (symbiotic) relationship. About 20,000 lichen species are known in the world, and there are approximately 700 species known from coastal rocks and urbanized shores [39]. Most work on aquatic lichens was done in temperate areas, as, in the tropics, lichens are less developed on costal rocks. One interesting skill is that lichen associations are primarily terrestrial but require alternate wetting and drying regimes for their survival. In marine environments, these circumstances occur principally in tidal zones on coastal rocks, subject to varying water levels and different degrees of inundation. Another feature is that the whole combined life form has properties that are very different from properties of its component organisms alone. Thus, tropical stream margins are promising biota for species and therefore compounds that are new to science.

### 2.3. Structural Diversity and Colors of Anthraquinoid Extrolites from Marine-Derived Fungi

#### 2.3.1. Present Knowledge about Anthraquinonoid Compounds from Fungi

Today′s knowledge indicates that a large part of compounds identified in terrestrial fungi can often be isolated from the same species living in marine environments. For instance, catenarin, emodin, erythroglaucin, physcion, questin, and rubrocristin or physcion anthrone are produced by marine-derived *Aspergillus* and/or *Eurotium* species, as well as by their terrestrial counterparts. According to Bick *et al.* and Fain *et al.* [40,41], the most widespread anthraquinones in fungi are 1,8-dihydroxy and 1,5,8 or 1,6,8-trihydroxy anthraquinone derivatives. They appear either as simple forms, as glycosides, or other complexes attached through an O- or C-bond in the side chain, which can enhance the water solubility. Some dimeric structures (formed through C–C bonds) are also produced from fungi, (e.g., alterporriols, skyrin, rubroskyrin, luteoskyrin, icterinoidin, rubellin, rufoolivacin, *etc.*). Some dimers may contain not only monomeric anthraquinones but also naphthoquinones and other products of polyketide synthesis. According to Fujitake *et al.* [42,43] and Suzuki *et al.* [44], the dimeric anthraquinone 5,5′-biphyscion (named hinakurin), chrysotalunin, (−)-7,7′-biphyscion, microcarpin, chrysophanol, and physcion are predominant in soil, but they seem rare in organisms. If hinakurin, chrysotalunin, and (−)-7,7′-biphyscion have not been found in fungi yet, it is now clear that chrysophanol and physcion are frequent fungal productions, and that fungi are able to synthesize dimers. These organisms, widely represented in soil, may have a transient appearance of monomeric and dimeric anthraquinones in telluric biotopes, certainly evolving to complex (humic) polymers. However, these statements rely on decades of terrestrial studies. The increase in knowledge on marine and marine-derived anthraquinones from fungi will certainly elucidate this aspect.

#### 2.3.2. Nature and Colors of Compounds from Marine-Derived Fungi

Most anthraquinoid compounds of natural origin have complex structures with several functional substituent groups. In nature and/or cultures, a wide range of hues appears, from pale yellow to dark brown, through to orange, red, or violet pigmentations. Anthraquinoid compounds generaly color sexual stages or resistance forms (ascomata, spores, conidia, *etc.*) but sometimes also impregnate mycelium or are excreted in the growth environment. Thus, the structural localization and the colors of these secondary metabolites seem to highly depend on the fungal species and may vary with the amount of compound produced in relation with the environmental conditions.

##### Genera and Species

Many genera producing anthraquinones have been isolated from marine environments, either from water, sediments, or decaying plants or from living organisms such as invertebrates, plants (endophytes), and algae. To date, strains in the genera—*Alternaria*, *Aspergillus*, *Eurotium*, *Fusarium*, *Halorosellinia*, *Microsphaeropsis*, *Monodictys*, *Nigrospora*, *Paecilomyces*, *Penicillium*, *Phomopsis*, and *Stemphylium—*have been clearly mentioned as marine-derived anthraquinones producers*.*

Some of the common terrestrial genera—*Aspergillus*, *Eurotium*, *Alternaria*, *Penicillium and Fusarium*—have been extensively investigated concerning their secondary metabolite′s productions, and the anthraquinoid molecules produced were reviewed by Velmurugan *et al.* [45], Caro *et al.* [16], and Gessler *et al.* [17]. They have revealed a large taxonomic distribution, confirming that the biosynthesis of anthraquinones is widespread in the fungal world, from macroscopic fungi to molds, as well as in lichens (symbiosis between algae and fungi). An extended list of the marine-derived fungal species producing anthraquinoid compounds with related differences is presented in Table 1. It indicates that, among the widespread compounds identified in marine-derived isolates, physcion, emodin, and chrysophanol and subsequently catenarin, erythroglaucin, macrosporin, and questin are frequently detected. The stuctural diversity of the compounds identified, along with the colors—(mentioned as a tag accompanying the molecule formula) are described in Table 2.

##### Ubiquitous Fungi

The Aspergillus glaucus group, A. variecolor, A. versicolor as well as Eurotium cristatum, *E. repens* and *E. rubrum*, very rife on earth, were also identified in marine niches.

To date, *Aspergillus glaucus* is the best anthraquinones producer from this *Aspergillus*/*Eurotium* cluster, according to the diversity of anthraquinoids molecules listed (11 different molecules from personal data [110]). Nine (9) isolates originate from marine or salted environments (sea, saltern, mangrove), suggesting a developed capacity to face high NaCl concentrations. These marine-derived isolates produce the main part of the new compounds identified in this genus up to now, besides endolichenic *Aspergilli*.

Catenarin, emodin, erythroglaucin, physcion, questin, and rubrocristin or physcion anthrone are commonly produced by marinederived *Aspergillus* and/or *Eurotium* species, as well as by their terrestrial counterparts. Moreover, Variecolorquinone A, which seems specific to the *Aspergillus* family, is synthesized by *Aspergillus* *glaucus* and *A. variecolor* B-17 from salted environments.

10,10′-dimer of emodin and physcion along with cynodontin, helminthosporin, tritisporin, unusual in this genus are excreted by the mangrove strain *A. glaucus* HB1-19. Two new hexahydroanthrones*—*tetrahydrobostrycin and 1-deoxytetrahydrobostrycin—are produced by *Aspergillus* sp. 05F16, a strain isolated from an unidentified alga collected in a coral reef (Indonesia).

The new methyl-emodin and 7-hydroxyemodin 6,8-methyl ether, along with emodin, were identified in the *A. versicolor* anendophytic strain isolated from the Red Sea green alga *Halimeda opuntia.* The new 6,8-di-*O*-methyl averantin, along with six known congeners are also synthesized by *A. versicolor* EN-7 (Genbank noEU042148*)*, an endophytic fungus of *Sargassum thumbergii* (brown algae).

The common genus *Eurotium* consists in teleomorphic, often xerophilic, species, usually related to *Aspergillus* anamorphs, especially from the *A. glaucus group.* Anke *et al.* [36] reported that, inside the *Eurotium* genus, *E. rubrum*, and *E. cristatum* produce the highest diversity of compounds; regarding anthraquinones, physcion, physcion anthrone, erythroglaucyn, catenarin, rubrocristin and emodin have been identified in their cultures. They also demonstrated that, inside a species, there was a great variability towards anthraquinones production, as some strains of *Eurotium* (among them *E. rubrum and E. herbariorum)* behaved differently in the same culture conditions. Moreover, some of the strains studied did not produce any anthraquinone, in the conditions of the experiment. Butinar *et al.* [111], noticed that, in Slovenian solar salterns, *E. amstellodami*, *E. herbariorum* and *E. repens* contributed to indigenous fungal community in hypersaline water environments, while *E. rubrum* and *E. chevaliery* were only temporal inhabitants of brine at lower salinities. In contrast, they stated that, for the six *Eurotium* strains isolated from these salterns, the qualitative secondary metabolites profiles were not different from those of strains isolated from foods or other habitats. However, the new 6,3-*O*-(α-d-ribofuranosyl) questin (anthraquinone gylycoside) is a new questin derivative from the marine-derived *E. rubrum* QEN-0407-G2, isolated from the marine mangrove plant *Hibiscus tiliaceus*. This compound, produced along with questin, seems unusual in the genus [63].

The unidentified fungus ZSUH-36 (isolated from the Shenzhen mangrove plant *Acanthus ilicifolius Linn.)*, the isolate 1850 (from a leaf of *Kandelia candel* from an estuarine mangrove in Hong Kong), and the mangrove endophytic strain 2526 produce versicolorin C, although the A enantiomer seems more common from the terrestrial producers *Aspergillus versicolor* and *A. parasiticus.* The two marine-derived strains, isolate 1850 and isolate 2526, also excrete the new nidurufin, along with the known averufin, also found in emerged *Aspergillus parasiticus* and *A.* *versicolor*. Fungus strain ZSUH-36 produces several new compounds, namely, 6,8-di-*O*-methyl averufin, 6,8-di-*O*-methyl averufanin, 1′-*O*-methyl averantin, and 6,8,1′-tri-*O*-methyl averantin.

*Penicillium* is a very widespread genus on earth and in marine biotopes. It seems to adjust easily to multiples conditions and to be a source of original compounds. The most commonly represented molecules in the terrestrial strains are emodin (and derivatives), rugulosin, and skyrin (or luteoskyrin), and then carviolin and chrysophanol (personal data, [110]). *Penicillium citrinum* PSU-F51 isolated from the gorgonian sea fan *Annella* sp. and *P. oxalicum* 2-HL-M-6 (from the sea mud sample) synthesize the common chrysophanol and emodin, but also citreorosein (ω-hydroxyemodin), a compound that, to date, is only isolated from the coprophilous ascomycete *Zopfiella longicaudata* IFM4630 [88,91]*.* Penicillanthranins A and B (two new anthraquinone-citrinin derivatives) are also excreted by *Penicillium citrinum* PSU-F51. Two new molecules—citreorosein-3-*O*-sulfate and emodin-3-*O*-sulfate as well as 1,8-dihydroxy-3-(hydroxymethyl) anthracene-9,10-dione (aloe emodin)—are engendered by *P. oxalicum* 2-HL-M-6. This strain also produces isorhodoptilometrin, which is uncommon, as it is only known from one plant-endophytic *Aspergillus* sp. (strain YL-6) [91,112]. *Penicillium chrysogenum* (from a saline lake in Antarctica) is the only aquatic fungus known for the production of skyrin, similarly to its terrestrial counterpart and four other terrestrial strains (*P. islandicum*, *Talaromyces wortmanii*, and *Dermocybe* spp.) [89]. In a related genera, *Paecilomyces* sp. (Tree 1-7*)*, a mangrove-derived fungus from a Taiwan strait, also produces chrysophanol and emodin [87].

##### Endophytes and/or Pathogens

*Trichoderma* is a frequent genus among vegetal root-associated fungi (endophytes and symbionts). Few marine-derived strains have been identified, to date, as anthraquinoid producers. However, the frequent emodin, ω-hydroxyemodin, pachybasin, ω-hydroxypachybasin, 1-hydroxy-3-methoxyanthraquinone, 2-methylquinizarin, and chrysophanol were detected in *Trichoderma aureoviride* PSU-F95, cultured from a gorgonian sea fan *Annella* sp. [88]. This strain also produces the new trichodermaquinone (tetrahydroanthraquinone), the rare coniothranthraquinone, or isorhodoptilometrin. *Trichoderma* strains from terrestrial origin rather produce chrysophanol and pachybasin (personal data, [110]).

Several species of *Alternaria* are terrestrial plants pathogens. Terrestrial *Alternaria* anthraquinones′ producers are found in many species, including *A. eichorniae,* also isolated from marine environments. The main represented compounds in this genus are macrosporin, altersolanol A, and 6-methylxanthopurpurin-3-*O*-methyl ether. However, original compounds were mainly isolated from marine-derived isolates. Indeed, alterporriols (A, B, C), altersolanol A, austrocortinin, bostrycin, physcion, and macrosporin are excreted by *Alternaria* species, originating from marine niches. Alterporriols C is commonly found in terrestrial and aquatic strains, but, until now, alterporriol A and B are only ones known from *Alternaria*′*s* marine-derived strains or from the terrestrial brother-group *Stemphylium* (*S. globuliferum*). This similarity in extrolite production with this genus also concerns altersolanol A, produced by several *Stemphylium* strains, from terrestrial or salted environments (*S. botryosum v. lactucum*, *S. globuliferum)*.

Concerning original compounds, *Alternaria eichorniae*, a pathogen of the water hyacinth *Eichhornia crassipes*, produces 4-deoxybostrycin, a rare compound only identified in a marine-derived *Nigrospora* sp. [46]. *Alternaria* sp. ZJ9-6B, a mangrove strain from the South China Sea, synthesizes seven new compounds: alterporriol K, L and M, dactylariol, alternariol (AOH), alternariol methyl ether (AME), and tetrahydroaltersolanol B [49]. The mangrove endophytic strain *Alternaria* sp. SK11, from the root of *Excoecaria agallocha* collected in the South China Sea, produces alterporriol S, (+)-aS-alterporriol C, 6-methylquinizarin along with the known austrocortinin [47]. *Alternaria* sp. ZJ-2008003, a fungus obtained from a *Sarcophyton* sp. soft coral from the South China Sea, is the only known strain to produce alterporriols N–R (five new alterporriol-type anthranoid dimers) [48].

*Stemphylium globuliferum* (from *Juncus acutus* collected from an hypersaline lake in Egypt) generates the common compounds alterporriol D and E, altersolanol A and B, and macrosporin, as well as the genus specific 6-*O*-methylalaternin [94]. Seven new compounds can also be found in this strain: altersolanol C, dihydroaltersolanol B and C, acetylalterporriols D and E (atropisomers), and stemphylanthranols A and B (the first naturally occurring trimeric anthraquinone derivatives). This endophytic/pathogenic fungus seems very productive in new compounds in marine environments. Indeed, the strain *Stemphylium* sp. 33231, obtained from the mangrove plant *Bruguiera sexangula var. rhynchopetala*, excreted four new alterporriol-type anthranoid dimers, along with 17 analogues [93]. In terrestrial habitats, the genus *Stemphylium* (anamorph of *Pleospora*) consists of plants pathogens/endophytes. The number of original molecules found in its productions is feeding the idea of originality in compounds coming from plant/microbe associations (alterporriol G and H (atropisomers), altersolanols K and L, as well as the new stemphypyrone) [113].

*Fusarium* is a widespread plant pathogen. The marine-derived *Fusarium* strains, mainly associated with marine organisms, produce four completely new anthraquinoid compounds. *Fusarium* sp. No. B77 (a mangrove endophytic strain from the south China sea) produces the new 5-acetyl-2-methoxy-1,4,6-trihydroxy-anthraquinone, and *F.* sp. ZZF60, another mangrove endophytic fungus from the same area, synthesizes the new 6,8-dimethoxy-1-methyl-2-(3-oxobutyl)-anthraquinone. The strain *F.* sp. No. ZH-210 coming from mangrove sediments of Zhuhai (China) produces the new fusaquinon B and C (red anthraquinone derivatives) along with the new fusaquinon A (colorless) [68]. *Fusarium* spp. PSU-F14 and PSU-F135 (endophytes from the gorgonian sea fan *Annella* sp., collected in Thailand) excrete the known bostrycin but also austrocortirubin, mainly known from the terrestrial macromycete *Cortinarius* spp.

*Microsphaeropsis* sp. (associated with the mediterranean sponge *Aplysina aerophoba*) produces 1,3,6,8-tetrahydroxyanthraquinone, also extracted from terrestrial *Geosmithia*, *Trichoderma*, and *Verticicladiella.* Three new C2-derivatives of 1,3,6,8-tetrahydroxyanthraquinone were also isolated from this marine derive fungi for the first time.

*Monodictys* (from a Japanese sea urchin *Anthocidaris crassipina*) is producing the common pachybasin, which was found for the first time from this species, along with emodin and chrysophanol, and also the new monodictyquinone A.

*Halorosellinia* sp. No. 1403 isolated from *Kandelia* sp. decayed woody tissue in Mai Po (Hong Kong, South China Sea) from a salt lake in the Bahamas excretes austrocortirubin, demethoxyaustrocortirubin, hydroxy-9,10-anthraquinone and two new compounds: 1,4,6-trihydroxy-2-methoxy-7-methylanthracene-9,10-dione and the patented 2,3,4,5,8,10-hexahydroxy-7-methoxy-3-methyl-1,3,4,10-tetrahydro-9(2*H*)-anthracenone (patented compound SZ-685C, [104]).

The endophytic *Nigrospora* sp. isolated from the mangrove plant *Bruguiera sexangula* is able to synthesize the new 1-deoxytetrahydrobostrycin (synonym: 8-hydroxytetrahydroaltersolanol B) along with bostrycin and 4-deoxybostrycin, depending on the culture media [83,114]. *Nigrospora* sp. 1403, endophytic from *Kandelia candel* in a marine mangrove (South China Sea), also produces bostrycin as two terrestrial strains [114,115] along with deoxybostrycin [86]. *Nigrospora* sp. isolated from an unidentified sea anemone excretes the new compounds 4a-*epi*-9α-methoxydihydrodeoxybostrycin and 10-deoxybostrycin along with seven known anthraquinone derivatives [84].

The new (2*R*,3*S*)-7-ethyl-1,2,3,4-tetrahydro-2,3,8-trihydroxy-6-methoxy-3-methyl-9,10-anthracenedione (new tetrahydroanthraquinone derivative) and the compound 1-hydroxy-3-methoxy-6-methylanthraquinone (first time isolation from fungi), along with macrosporin and tetrahydroaltersolanol B&C, were extracted from a culture of *Phomopsis* sp. strain (PSU-MA214*)* isolated from a leaf of a mangrove plant *Rhizophora apiculata*. This is different from a terrestrial strain of *P. juniperovora* (PM0409092) producing the common altersolanol A.

*Curvularia lunata* (anamorphic stage of *Cochliobolus lunatus*), isolated from the marine sponge *Niphates olemda* in Indonesia, excretes the new lunatin and cytoskyrin A, a molecule only known from one another species *Cytospora* sp. CR 200 (endophyte from the buttonwood tree *Conocarpus erecta* in Costa Rica) [60].

Reviewed by Crous *et al.* [116], many species of *Arthinium* are associated with plants as endophytes or parasites, even in marine conditions. Somes strains of *Athrinium phaeospermum* cause cutaneous infections of humans. Others are involved in endophytic plant relationships, producing growth promoting substances, e.g., in *Carex kobomugi*. The endophytic *Arthrinium* *phaeospermum CBS 142.55* (type strain of *Botryoconis sanguinea*) produced bostrycin with some minor unidentified red and yellow pigments when cultured on malt extract agar [117]. Bostrycin is produced by *Bostrychonema alpestre*, the causal agent of water hyacinth blight disease, as well as by other plant pathogenic fungi such as *Alternaria eichorniae*, *Nigrospora oryzae*, *Arthrinium phaeospermum* and *Fusarium* spp. *Bostrychonema alpestre* (terrestrial strains) also excretes austrocortilutein and torosachrysone mainly found in macrofungi as *Dermocybe splendida* [118].

As far as bostrycin is concerned, the originally proposed structure [119,120,121] was revised by Kelly and his co-workers in 1985 [122] on the basis of the total synthesis of (+/−)-bostrycin and an X-ray crystal structure of the *O*-isopropylidene derivative. This revision also concerns its derivative 4-deoxybostrycin found in serveral marine isolates [68,84,86,101,123].

A marine-derived strain of *Xylaria* sp. 2508 produces a new compound, xylanthraquinone, along with three known anthraquinones, altersolanol A, deoxybostrycin, and bostrycin [95]. The *Xylariaceae* is one of the largest families of endophytic filamentous fungi isolated from plants material in terrestrial biotopes. There, they essentially grow under the form of mycelial structure, and their fruiting bodies (stromata) seem to form only when their host is stressed or diseased. However, the pigments seem to be mainly extracted from their fruiting stages [124]. Under marine conditions the morphological structures of fungi are not yet precisely described, and researchers suppose that fungi are mainly growing under mycelial structures. Nevertheless, this does not prohibit the synthesis of these anthraquinoid molecules.

To date, over 100 different anthraquinoid metabolites have been identified in around 27 marine-derived fungal isolates, belonging to at least 22 identified species.

##### Lichens

*Caloplaca*, *Collema*, *Collemopsidium*, *Lichina*, *Ochrolechia*, *Ramalina*, *Tephromela*, *Verrucaria,* and *Xanthoria* species are frequent among lichenic maritime populations [125]. They are known, in marine as in terrestrial biota, to be frequent producers of the common physcion (parietin). This anthraquinoid compound is excreted by *Xanthoria aureola* and *X. parietina* collected from exposed maritime rocks (South Norway) [126,127]. A high diversity of specific compounds can, however, be obtained from lichens, particularly in species producing chlorinated anthraquinones [76,96,128]. The production of special anthraquinones is a major characteristic of most species in the family *Teloschistaceae* (*Caloplaca*, *Xanthoria, etc.*) [72,77]. In the genus *Xanthoria*, and in closely related species of *Caloplaca*, antraquinoid molecules seem to be the only lichenic secondary compounds present [129,130]). The majority of species produce the widespread parietin and its chemical relatives, but also fragilin, emodin, and chloroemodin, accompanied by varying amounts of their oxydation products [128,129,131]. *Caloplaca* spp. isolated from calciferous rocks in Central Asia (*C. schaereri*; *C. spitsbergensis*, *C. ehrenbergii,* and other species) effectively produce 7-chlorocitreorosein and 7-chloro-1,6,8-trihydroxy-3-methyl-10-anthrone, as well as 7-chloroemodic acid, 7-chloroemodinal, and 1-*O*-methyl-7-chloroemodin, but also fallacinol (teloschistin) and fallacinal. Many of these compounds, widespread among lichens, are not found outside from the lichen′s group.

Some fungal mycobiontes involved in lichen associations also proved their capacity to produce several original anthraquinoid molecules in separate cultures. For instance, *Aspergillus versicolor*, a mycobionte in *Lobaria retigera* excretes averythin, 8-*O*-methylaverythin, 8-*O*-methylversicolorin A and B, 6,8-di-*O*-methylversicolorin A, 6,8-di-*O*-methylaverufin, and 6,8,1′-tri-*O*-methylaverantin [108].

## 3. Biosynthesis and Known Roles for Anthraquinones in Fungi

The metabolism is the sum of all the biochemical reactions carried out by an organism. Opposite to the primary metabolism, converging to few products common to many organisms, the secondary metabolic pathways diverge to a great diversity of molecules produced from a few key intermediates of primary metabolism. While endometabolites can be found in almost all species of fungi, exometabolites seem taxonomically shared by species–specific profiles [132,133]. Some authors then assert that the nature of the extrolites can act as signatures, specific to the organisms, coming from biogenetics patterns. Thereby, in *Dermocybe* spp., it was possible to group some subsections with regards to biosynthetis of particular anthraquinoid compounds (skyrin, icterinoidin or hypericin) [134]. Considering the significant shortcomings in these research fields, future studies will gain knowledge about the truth of these claims. Undoubtedly subservient to the genetic skills, the biosynthesis of metabolites such as anthraquinone derivatives is clearly influenced and regulated by a complex set of factors including biotic and abiotic dimensions. Such parameters as temperature, pH, [O_2_] availability, light exposure, nature, and abundance of nutritive sources, as well as age and specialization of the fungal structures, have already been proven to have a strong influence on pigments production [135,136,137,138,139].

### 3.1. Biosynthetic Route and Genes Involved

As polyketides seem to be the most abundant extrolites in fungi, the pathways for their biosynthesis have been widely explored [140]. However, the formation of anthraquinones in fungi is not the most developed area in the literature.

**Figure 3:**
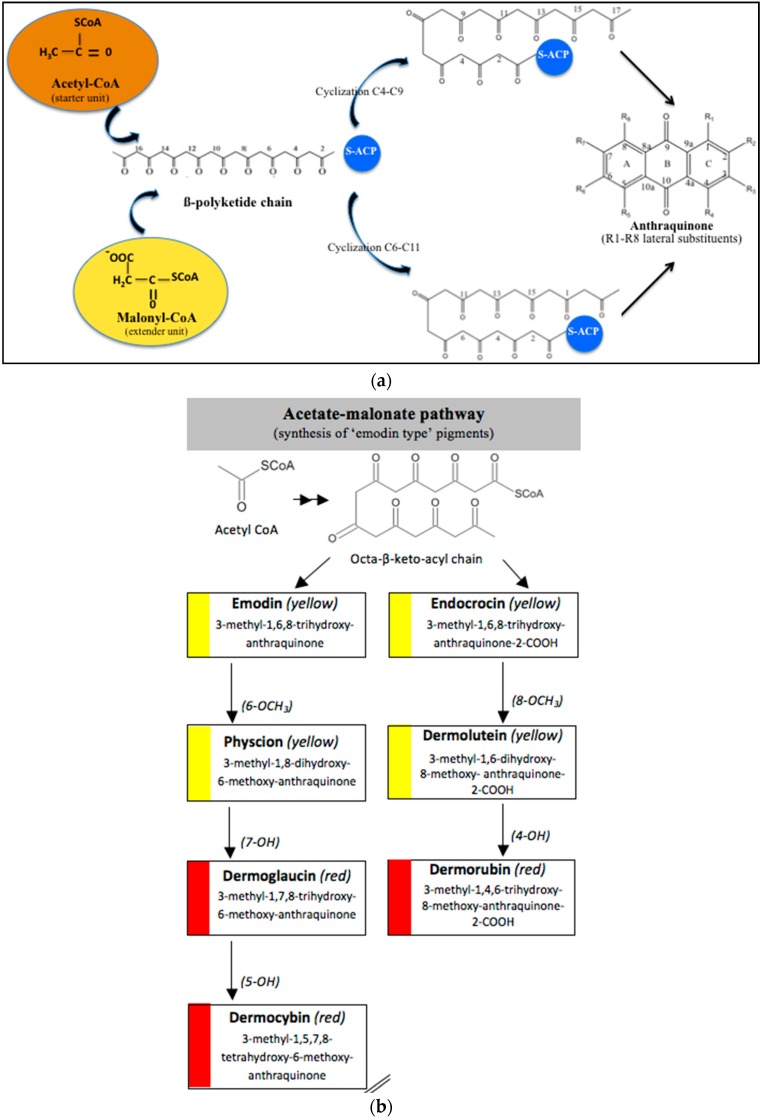
Anthraquinones biosynthetic pathway in fungi. (**a**) Regioselectivity in the formation of the β-polyketide chain during the synthesis of antraquinones in fungi (adapted from Gessler *et al.* [17]). ACP: Acyl Carrier Protein; (**b**) Anthraquinones acetate-malonate pathway in fungi: synthesis of emodin type pigments (from Caro *et al.* [16]).

In fungi polyketide compounds are primarily synthesized by the acetate-malonate pathway (Figure 3), which is different from plants using shikimate pathways as well as acetate-malonate metabolism. Depending on the presence of acetate, malonate, or both components, according to the growth conditions, and to the strains involved, the numbers of each residue incorporated differ, and the final secondary metabolites as well. The involvement of each type of molecule can be studied through experiments using [^13−14^C-acetate] and [^13−14^C-malonate], ([141] and other authors such as Gessler *et al.* [17]. In relation to this acetate-malonate pathway, the biosynthetic relationships seem to form yellow hydroxyanthraquinones (e.g., emodin, physcion, and dermolutein) at the beginning of the pathway (simple structures), whereas the red ones, such as dermorubin or dermocybin, with more complex structures, certainly occur in the latter part of the biosynthesis pathway [16].

Hanson *et al.* [15] and Gessler *et al.* [17] summarized that anthraquinones synthesis are regulated by non-reducing polyketide synthases (NR-PKS′s). These multidomains enzymes mediate the regioselective cyclization of polyketides, clearly dominating the final structures. NR-PKS′s form polyketides in which carbonyl groups are not reduced, whereas reducing polyketide synthases partially or fully reduces the carbonyl groups [142,143]. These multifunction complexes, including acyl carrier protein (ACP), transacylase (STA), ketosynthase (KS), malonyl-CoA transacylase (MTA), thioesterase (TE), product template (PT) domain, methyltransferases, and reductases, first ensure the condensation of acetyl-CoA (starter unit) and malonyl-CoA (extender unit). This produces an instable β-polyketide chain (containing a free carboxylate group) precursor of different aromatic structures. There are several types of PKSs found in different organisms (groups I-VIII), but the principles of constructing the poly-β-keto chain are the same with all PKSs.

From these, the fungal PKS are of considerable interest due to their interesting enzymology and the final polyketide structural diversity [144]. One of the earlier major advances in identification of fungal polyketide secondary metabolite gene clusters is the development of a degenerate-primed polymerase chain reaction (PCR), based on the conserved ketosynthase domain of PKS [145]. Javidpour *et al.* [146] asserted that the specific enzyme product template (PT) domain, determined the regioselectivity of the cyclization of the polyketide chain, and then the final structure of the products. Thus, they appeared as key factors in the biodiversity of these secondary metabolites. In fungi PT, domains are found in all 8 groups, but anthraquinoid compounds seem to be mainly produced from PT IV and V groups, indicating C4-C9 or C6-C11 cyclizations.

Based on a bioinformatical analysis, Liu *et al.* [147] interestingly clarified the relationships between enzyme sequences, structures, and functions in fungal PKS PT domains. Besides the basic PKS domain, additional functional domains, including the SAT (starter unit-ACP transacylase) domain, the PT (product template) domain, and the TE (thioesterase) releasing domain, are unique to the NR-PKSs. The PT domains have been demonstrated to be involved in controlling specific aldol cyclization and aromatization of the polyketide precursors. For the first cyclization, three commonly cyclizing patterns appear (C2-C7, C4-C9, and C6-C11). The comparison of 661 NR-PKS sequences belonging to ascomycota and basidimycota revealed that the PT domains can be classified into prominent eight groups (I–VIII) corresponding with the representative compounds and the cyclization regioselectivity. Most of the cavity lining residue (CLR) sites were common in all groups, while the regional CLR site mutations resulted in the appearance of finger-like regions with different orientation. The conservative residues in PT sequences were responsible for the cyclization functions and the evolution of the key residues resulted in the differentiations of cyclization functions. Thus, the cavity volumes and shapes, even the catalytic dyad positions of PT domains in different groups, corresponded to characteristic cyclization regioselectivity and compound sizes.

These authors also noticed that the cyclization route of the polyketide chains even differ between actinomycetes and fungi, certainly due to the involvment of different groups of PT enzymes [148]). Moreover, the late steps of the biosynthesis are responsible for the additions and deletions of lateral substituent groups, generating a great diversity of compounds. For instance, they assert that methyl groups of anthraquinones come from methionine residues via S-adenosylmethionine [149,150,151,152,153].

Miethbauer *et al.* [141] tried to elucidate the biosynthetic pathway of a complex anthraquinone via the synthesis of rubellins. Nineteen species of *Ramullaria* coming from different regions were tested for the production of rubellins A, B, C, D, E, and F. Seventeen biosynthesized the entire panel of rubellins, more or less intensively, except two of them: *R. pratensis and R. inaequalis*. They stated that, using [^13^C-acetate], rubellins are naturally synthesized through the polyketide pathway. They demonstrated that the methyl group of acetate must be converted in part to a carboxyl one via protein turnover, or more specifically by biosynthesis, and the subsequent degradation of lysine in fungi [154]. They suggested that rubellin A and B are certainly originating from a dimeric anthraquinone and that helminthosporin (1,5,8-trihydroxy-3-methylanthraquinone) can be considered the primordial monomer. The conversion of the keto group into a lactone is supposed to be carried out by a Baeyer-Villiger monooxygenase [155]. Thus, they proved that, if the majority of the strains can use these rubellins as nonspecific toxins against plants, some of the species are, surprisingly, completely unable to produce such molecules.

The recent whole-genome sequencing of various fungi revealed that these microorganisms have immense biosynthetic potential, surpassing, by far, the chemical diversity observed in laboratory cultures. For example, the genome of many *Aspergilli* encodes a combined 30 to 80 PKS—non-ribosomal peptide synthetases and polyketide non-ribosomal peptide synthetases hybrids—which far exceed the total number of known polyketides and non-ribosomal peptides [156].

Recently, Bringmann *et al.* [157] revealed that the pigment chrysophanol can come from an organism-specific route, through a third folding mode involving a remarkable cyclization of a bicyclic diketo precursor. This establishes the first example of multiple convergences in polyketide biosynthesis. The programming of the fungal PKS seems quite complex and suitable to form sophisticated products. An additional level of complexity can be imagined combinatorializing PKS-based pathways with other metabolic routes.

Kakule *et al.* [158] realized a gene fusions with the idea of testing the connection and compatibility of the PKS and NRPS (nonribosomal peptide synthetase) modules, mediated by the ACP, condensation (C) and ketoreductase (KR) domains. The resulting recombinant gene fusions availed six new compounds. They obtained the first successful fusion between a PKS and NRPS that make highly divergent products as well as previously reported molecules. Thus, they demonstrated that, within the highly reducing (hr) PKS class, noncognate ACPs of closely related members can complement PKS function.

Today, it is rather clear that many of the genes involved in the polyketide pathway are organized in gene clusters, which are often silent or barely expressed under laboratory conditions. This makes their study more difficult. Fortunately, the genome sequences of several filamentous fungi are now publicly available, greatly facilitating the establishment of links between genes and metabolites. To date, complete knowledge about the biosynthetic pathway of hydroxyanthraquinonoid compounds is not yet available, but this knowledge is increasing daily.

### 3.2. Roles in the Biology of Fungi

Anthraquinones belong to secondary metabolites. The secondary metabolic pathways are today considered as diversions from the primary metabolism, leading to a great diversity of compounds, arising from a few key intermediates. Secondary metabolites, also known as exometabolites, are produced during morphological and chemical differentiation, either accumulated or excreted. In contrast, endometabolites (primary metabolites) fluctuate in concentrations and either transform into other endometabolites or feed into exometabolites. Secondary metabolites are bound to appear when the environmental conditions become unfavorable, in particular when a substrate other than carbon becomes limited [159]. This process of derivation is important in itself. Indeed it is supposed to provide a route for the removal of intermediates, which would otherwise accumulate. This accumulation could probably enable the primary processes leading to these intermediates to remain operational during the time of stress [160]. For researchers, this formerly implied two main features: (1) Cells that are no longer undergoing balanced growth synthesize them for a finite period; and (2) no obvious functions were clearly demonstrated in cell growth for these compounds. Thus, they appeared to researchers to be not vital to the cell life itself. Nowadays, the trend is, through their direct or indirect actions, secondary metabolites are important for the entire organism′s survival [161,162,163]. Due to their chemical and biological properties they are today considered as first-plan actors to help in protection, competition, symbiosis, metal transport, differentiation, *etc.* The biological significance of anthraquinoids pigments, as others such as carotenoids, involves resistance to a variety of adverse environmental factors (dessication, exposure at extreme temperatures, irradiations), to antimicrobial activity (antibacterial, antiviral). Many observations have now been published with regard to the antioxidant activity of carotenoids pigments, protective action against lethal photooxydation, inhibition of mutagenesis, enhancement of the immune response, and inhibition of tumor development [164]. Melanin was also suggested to act as a shield against immunologically active cells [165].

Besides the intensively investigated fungal azaphilone pigments, anthraquinone compounds have been considered among the most abundant fungal natural products, giving color to spores, sclerotia, sexual bodies, and other developmental structures [139]. Present research suggests that, in specific cases, it is doubtful that pigments are really secondary metabolites [164]. It is now clear that anthraquinones should play a role in the cell life.

#### 3.2.1. Anti-Oxidant/Pro-oxidant Activities

Solar radiations contain stress factors for living organisms, especially UV-B. Thus, the biological role of pigments, as the dark brown to black melanin and melanin-like pigments, often restricted to certain developmental stages or special structures (chlamydospores or microsclerotia), is clearly dedicated to growth, dissemination, and survival under unfavorable conditions. The accumulation of pigment acts as a radiation-screening system that prevents the occurence of damages. It is clear that, in unusual biotopes (sometimes extreme), the fungal species with pigmented cell walls (in the sexual stages, conidia, and/or mycelium) are able to tolerate dehydration-hydration cycles or high solar radiations better than the moniliaceous fungi, whose cells are devoid of pigments. These aromatic compounds, such as melanin, sporopollenin (brown product of oxidative polymerization of β-carotene) and cycloleucomelone (terphenylquinone) [18], often show significant antioxidant activities and are bound to protect the biological structures, giving them an excellent durability and a high potential for survival in hostile environments. However, at higher doses or under certain conditions, antioxidant-type functional compounds may exert toxic pro-oxidant activities, as was demonstrated *in vitro* for vitamin C, E, carotenoids or flavonoids [166]. This is not a new idea, as Paracelsus (1493–1541) already reported toxicity to be a matter of dose. Thus, toxicological risks may arise when daily doses of a compound rise above a certain threshold limit.

Things are not so clear about the relationships between carcinogenic effect and pro-oxidant properties. Indeed, pro-oxidant natural products inducing ROS (reactive oxygen species) were proved to contribute to anticancer effects as for campothecin derivatives [167]. Pro-oxidant natural products then become an attractive anticancer strategy.

Identically, quinonoid structures allow anthraquinones to participate in redox reactions, exhibiting antioxidant or pro-oxidant properties. Emodin and physcion have clearly demonstrated antioxidant and antimicrobial activities [168]. According to Yen *et al.* [169], the basic anthrone chemical structure exhibited the role of electron acceptor, and the ortho-dihydroxy substituent in alizarin, the polyhydroxyl group at position C1, C6 and C8 with methylation at position C3 (emodin), and the polyhydroxyl group at position C1 and C8 with hydroxylmethylation at position C3 (aloe-emodin), are multifunctional antioxidants, combining both chain-breaking and metal-chelating properties. Moreover, they stated that the greater reducing power and the metal chelating activity of alizarin may relate to its marked antioxidant activity. On the other hand, the significant scavenging effect of emodin and aloe-emodin on hydroxyl radicals may contribute to their antioxidant activity. Alaternin also inhibited the peroxidation of linoleic acid by the thiocyanate method in a dose-dependent manner, and showed inhibitory activities in reactive oxygen- and nitrogen-mediated reactions, indicating that it is a potentially effective and versatile antioxidant useful, for protecting biological systems and functions against various oxidative stresses [170]. On the other hand, chrysophanol accelerated the peroxidation of linoleic acid.

Another illustration is the symbiotic foliose lichen *Xanthoria parietina*, which is able to colonize very extreme environments. The cortical anthraquinoid pigment parietin (physcion) is mainly synthesized by the mycobionte and absorbs UV-B and part of PAR (photosynthetically active radiations). Its production depends on several factors. The UV-B induction (280–320nm) and the photosynthetic assimilation of carbon in the photobionte, the green algae *Trebouxia arboricola*, increases the production of parietin; the lack of PAR, dessication, or substancial depression decreases the production [171,172]. Conversely, in artificial culture conditions, it seems that light in the visible part of the spectrum significantly decreases the pigment production in *Isaria farinosa*, *E. nidulans*, *F. verticilloides*, and *P. purpurogenum* [173].

There is therefore a strong correlation between the anthraquinoid contents and site factors, including the openness of the habitat, suggesting that solar radiation plays a crucial role.

#### 3.2.2. Competition and Symbiosis

Endophytic/symbiotic associations may clearly involve some complex regulation mechanisms. Cases of interspecific regulations are already recorded concerning anthraquinones. *In vitro*, anthraquinones production in *Aspergillus* *kanagawaensis* is, for instance, stimulated by co-cultures with different species [174]. The study also proved that a hydroxyanthraquinone molecule was able to stimulate the synthesis of exoproteases, in *Aspergillus* *kanagawaensis* cultures alone and during co-culture with *A. wentii*.

*Trichoderma* is a frequent genus among vegetal roots associated fungi (endophytes, symbionts) [175]. By colonizing the surface of pinaceae roots, *Trichoderma viride*, for instance, is supposed to confer physiological benefits to the seedlings, enhancing the plant-soil exchanges and probably exerting a photoprotective activity [176]. From this review, this genus is also mentioned to suppress fungal disease in plants and soils, and also to promote plant growth [177]. The frequent antimicrobial activity of anthraquinones clearly enhances the host plant resistance against pathogens (bacteria, fungi). This led, for instance, to a patent describing a plant protection product, using a formulated Polygonaceae extract (*Reynoutria sachalinensis)* marketed as REGALIA^®^ SC by Marrone Bio Innovations, Inc. (Davis, CA, USA) [178]. The MBI-106 active principle contains at least physcion, emodin, chrysophanol, ventiloquinone, emodin glycoside, chrysophanol glycoside, physcion-glycoside, 3,4-dihydroxy-1-methoxyanthraquinone-2-corboxaldehyde, and damnacanthal. The results support a general hypothesis that the presence of the functional metabolites of endophytes enhances the host plant fitness and competitivness in stressful environments [179]. Moreover, they certainly play an important part in inter- or intra-specific competions, combating other microbes in natural micro-environments.

From a functional point of view, in the review of Gessler [17], catenarin and emodin were able to inhibit the DNA-dependant RNA-polymerase of *E. coli.* In *Trichoderma*, the increase in excreted emodin or pachybasin concentrations increases the concentration of cAMP, indicating that these compounds are key substances in the regulation of this secondary messenger and thereby in intracellular signaling. Alterporriol-type dimers from *S. globuliferum* and altersolanol from the endophytic *Ampelomyces* sp., as well as many other anthraquinones, are able to inhibit several protein kinases, playing significant roles in host cells proliferation (PKC-e, CDK4, EGF, *etc.*). The anticancer properties of some anthraquinones can also be partially explained through the inhibition of the Cdc25 phosphatase, demonstrated for emodin, questin, and physcion, as well as through the capacity to activate the capsase’s cascade, inducing apoptosis of tumor cells.

The vast array of metabolites produced by microbes in their growth environments undoubtedly has an ecological role in regulating the interactions between plants, microorganisms, insects, and animals. Highlighting these facts, we should ask ourselves about the high capacity of endophytes or symbionts to synthesize original molecules arising from the interactions with their host organisms. Many questions then arise about the contribution of partners in such associations, especially concerning anthraquinoid compounds.

#### 3.2.3. Chelating Properties

Greenaway *et al.* [180] proved that the red anthraquinoid pigment from *Pyrenophora avenae* (1,4,5,8-tetrahydroxyanthraquinone) removed phenyl-Hg^+^ ions (Phenyl Mercuric Acetate (PMA)) from an aqueous solution. They stated that strains secreting a visible amount of the pigment were invariably resistant to PMA, and those lacking the pigmentation were susceptible. They noticed that resistance to PMA and pigment production were linked in the segregation of resistant and susceptible progeny of conidiospore from a resistant colony. They concluded that the chelation of the Hg ions by the anthraquinone molecule produces a stable and non-toxigenic complex, protecting the cells proteins from binding with toxic mercury through their sulfhydryl groups. This demonstrated the detoxification potential of anthraquinoid pigments in strain metabolism. However, they demonstrated that pigment-producing strains grew more slowly than unpigmented ones in media lacking PMA. They suggested that an unsaturated chelator produced by resistant strains could act to their detriment by chelating substances required for growth. Several articles refer to the metal-chelating properties of anthraquinoid compounds *in vitro* (such as copper and palladium [181]). These favorable chelating properties towards toxic ions such as aluminium were also demonstrated in plants by studying the impact of soil anthraquinones on the growth of *Alfalfa* and lettuces [182]. However, they stated that the chelating properties were depending on the structure: Some anthraquinones presented an inactive form (glycoside form), while others demonstrated effective chelating properties (mainly monomeric: physcion, emodin, and chrysotalunin).

## 4. *In Vitro* Biological Activities of Natural Anthraquinones from Marine-Derived Filamentous Fungi

In recent years, marine microorganisms are increasingly attracting attention of the pharmaceutical community as they produce a wide variety of secondary metabolites that are structurally unique and pharmacologically active [104]. Naturally occurring anthraquinones have been recognized to possess anti-inflammatory, anti-fibrotic, and antitumor properties and thus are applied in human and veterinary therapeutics as active substances of medicinal products. Huang *et al.* [183] demonstrated that anthraquinones isolated from plants, such as emodin, aloe-emodin, and rhein, inhibit the growth and proliferation of various cancer cells, such as lung adenocarcinoma, myelogenous leukemia, neuroblastoma, hepatocellular carcinoma, bladder cancer, and others. The chemical diversity and biological activities of fungal anthraquinone and derivatives from marine origin are considerable; thus, they may represent a promising resource for identifying new therapeutic drugs candidates [184].

### 4.1. Antitumor Activity and Cytotoxicity

Cancer has become an increasing public health problem due to its high rates of morbidity and mortality. Conventional cancer chemotherapy has the limitation of multidrug resistance caused by an overexpression of integral membrane transporters, which can efflux intracellular anticancer drugs, thus decreasing drug accumulation. Clinical multidrug-resistant (MDR) cells are resistant to cytotoxic effects of various structurally and mechanistically unrelated chemotherapeutic agents. Developing new anticancer drugs that are efficient to MDR cells is a feasible strategy to overcome MDR. In the discovery of new fungal anthraquinone-derivative therapeutic molecules, cytotoxic activity is one of the prominent assays to develop novel anticancer drug candidates [184].

Cancer development largely results from uncontrolled growth of malignant cells in which cell proliferation surpasses cell death. Deregulation of apoptosis, occurring in a majority of cancer types, has since become a non-negligible target for anticancer strategies. Pro-apoptotic compounds derived from marine sources, are thus under active investigation for their therapeutic effects, and for their mode of actions against various cancers as well [104].

Apoptotic cells are usually characterized by several distinctive morphological and biochemical changes, including cell shrinkage, chromatin condensation, presence of phosphatidylserine on cell membrane surface, DNA fragmentation, protein cleavage at specific locations, and increased mitochondrial membrane permeability. A cascade of caspases activation in an ordered and regulated manner is also known to be involved in apotosis as a suicidal process [104].

#### 4.1.1. Breaking the Cell Cycle/Apoptosis Hallmarks

Norsolorinic acid, a fungal anthraquinone derivative originally isolated from the fungus *Aspergillus nidulans*, was investigated for its anti-proliferative activity in human breast adenocarcinoma MCF-7 cells by Wang *et al.* [185]. The results showed that it induced apoptosis of MCF-7 cells.

Using the yeast *Saccharomyces cerevisiae* as a model, Xu *et al.* [103] showed that bostrycin inhibits cell proliferation by blocking the cell cycle at G1 phase. Bostrycin-induced lethal cytotoxicity is actually accompanied with increased levels of intracellular reactive oxygen species and hallmarks of apoptosis such as chromatin condensation, DNA fragmentation, and externalization of phosphatidylserine. This compound also decreases mitochondrial membrane electric potential and causes mitochondrial destruction during the progression of cell death. In this case, apoptosis appears as a mitochondria-mediated but caspase-independent pathway.

In 2011, Ren *et al.* [186] reported the isolation of nidurufin, a hydroxy derivative of averufin, from the marine-derived fungus *Penicillium flavidorsum* SHK1-27. An evaluation of the antitumor activity indicated that nidurufin induced *in vitro* cell cycle arrest at G2/M transition in the leukemia K562 cell line, in a concentration and time-dependent manner, with an IC_50_ value of 12.6 μM.

Research findings on emodin-induced cytotoxicity and its protective effects against cancer in different body systems, as well as the antitumor mechanisms involved, are well documented in the literature, though still in progress. This compound (1,3,8-trihydroxy-6-methyl-anthraquinone) is reported to have multiple biological activities including antimicrobial, antiviral, anti-inflammatory, anti-ulcerogenic, immunosuppressive, chemo-preventive, and antitumor functions on digestive, respiratory, reproductive, and blood system cancers. It was first assigned to be a specific inhibitor of the protein tyrosine kinase p65lck. Its inhibitory effect on mammalian cell cycle modulation studied in specific oncogene overexpressed cells formed the basis of using this compound as an anticancer agent [187].

Recently, quinofuracins A–E, novel anthraquinone derivatives containing β-d-galactofuranose that were isolated from the fungus *Staphylotrichum boninense* PF1444, also induced p53-dependent cell death in human glioblastoma LNZTA3 cells [188].

#### 4.1.2. Deregulation of ALAS2/c-KIT/miR-221, miR-222, miR-200c, miR-205/Akt

Other pharmaceutical studies have shown that emodin may induce apoptosis but also reverse multidrug resistance in HL-60 and HL-60/ADR cells. It may improve the expression of globin genes in leukemia K562 cells. It also induces K562 cells to erythroid differentiation, possibly *via* upregulating ALAS2 and c-KIT, and downregulating miR-221 and miR-222 [189]. Furthermore, Chen *et al.* [190] reported that emodin exhibited significant anti-leukemic effects *in vitro*. Increasing a dose of emodin could effectively induce growth inhibition and apoptotic effects in NB4 and MR2 cell lines, as well as in primary leukemic cells from acute myeloid leukemia (AML) patients in a dose-dependent manner. This clonal hematopoietic stem cell disorder is characterized by differentiation arrest, inappropriate proliferation, and survival of immature myeloid progenitors. Importantly, emodin was demonstrated as a new inhibitor of phosphatidylinositol 3-kinase (PI3K)/Akt in AML cells. Indeed, cell death and survival are under modulation of a network of transmembrane and/or intracellular signals in which the PI3K/Akt pathway is a prominent mediator of the survival and the proliferation. PI3K/Akt signaling pathway is frequently activated in AML cells and in other malignant phenotypes in a wide variety of cancers. Akt′s phosphorylation occurs in response to PI3K activation. Thus, the interruption of the PI3K/Akt signaling pathway should be considered when designing anti-AML therapeutic strategies [190]. Emodin inhibited Akt phosphorylation (p-Akt) at Ser473 as efficiently as mTOR at Ser2448. Consistently, it exerted suppression effects on the phosphorylation of mTOR downstream targets, *i.e.*, 4E-BP1 and p70S6K. Therefore, they provided a demonstration that nontoxic dose of emodin inhibited growth and induced differentiation to a low degree in NB4 and MR2 cells. Moreover, the apoptotic induction in AML cells was associated with the activation of caspase cascades, involving caspase-9, caspase-3, and poly(ADP-ribose) polymerase cleavage. Taken together, these findings indicate that emodin might be considered a promising anti-leukemic agent to overcome all-*trans* retinoic acid-resistance and to improve the patient outcome in AML.

In this topic, previous studies reported the isolation of five anthraquinone derivatives from the marine endophytic fungus *Halorosellinia* sp. No. 1403 [70]. One of these compounds, e.g., the marine anthraquinone called SZ-685C showed strong cytotoxicity toward KB and KBv200 cancer cell lines, with IC_50_ values of 1.40 and 2.58 μg/mL, respectively [70]. This compound inhibits the growth of six tumor cell lines, including human glioma, hepatoma, prostate cancer, MCF-7, and MDA-MB-435 breast cancer cell lines, with IC_50_ < 10 μM [104]. Furthermore, *in vivo* experiments showed that SZ-685C inhibits the tumor growth in nude mice by inducing apoptosis via the Akt/forkhead box protein pathway [104]. Subsequently, Zhu *et al.* [106] found that this marine anthraquinone derivative causes apoptosis in adriamycin-resistant human breast cancer cells, both *in vitro* and *in vivo*. It exerts these antitumor effects through multiple mechanisms mainly involving the suppression of Akt signaling. More recently, Chen *et al.* [191] reported that SZ-685C significantly inhibited the proliferation of MMQ pituitary adenoma cells and induced apoptosis by downregulation of miR-200c. In addition, this compound showed potent anticancer activity in radiosensitive and radioresistant nasopharyngeal carcinoma cells, and the miR-205-PTEN-Akt pathway is the mechanism underlying the anticancer activity [192].

#### 4.1.3. Capsase Dependant Pathway Disturbance/Topoisomerase Inhibition

In 2013, Teiten *et al.* [193] reported that altersolanol A, a natural anthraquinone derivative originally isolated from the endophytic fungus *Stemphylium globuliferum*, showed cytotoxic, cytostatic, anti-inflammatory, and anti-migrative activity against human cancer cell lines (chronic myeloid K562 leukemia and A549 lung cancer cells) in a dose-dependent manner. Interestingly, this compound did not affect the viability of non-cancerous cells. Results clearly demonstrated that altersolanol A induces cell death by apoptosis through the cleavage of caspase-3 and -9, and through the decrease of antiapoptotic protein expression. Acetylation of altersolanol A did not improve activity, whereas other altersolanol derivatives such as tetrahydroaltersolanol B and ampelanol (one of the carbonyl group reduced and some hydroxyl substituents removed) were inactive in comparison.

More recently, a novel anthraquinone derivative with a complex skeleton, naphtho[1,2,3-*de*]chromene-2,7-dione skeleton, identified as aspergiolide A, was isolated from the marine-derived fungus *Aspergillus glaucus* [194]. The compound exhibited distinct cytotoxicities against cancer cell lines A-549, HL-60, BEL-7402, and P388. It was demonstrated that aspergiolide A had anticancer activity targeting topoisomerase II. It clearly decreased the growth of various human cancer cells *in vitro* and induced apoptosis in BEL-7402 cells via a caspase-dependent pathway. *In vivo*, aspergiolide A exhibited significant anticancer activity on the growth of hepatocellular carcinoma xenografts. The maximal tolerable dose of aspergiolide A was more than 400 mg/kg, and it was not considered to be potentially genotoxic or cardiotoxic.

Finally, the marine anthraquinone compound called G503 was isolated in 2014 from the secondary metabolites of the mangrove endophytic fungus *Halorosellinia* sp. No. 1403. This anthraquinone derivative was reported to have antitumor activity [195]. The experiments suggested that the intrinsic mitochondrial apoptosis pathway was also involved in G503-induced apoptosis. The endoplasmic reticulum apoptosis pathway might also be activated by G503 by inducing capase-4 cleavage. In consideration of this inhibition effect on gastric cancer cells, the marine anthraquinone derivative G503 may serve as a promising candidate for gastric cancer chemotherapy.

#### 4.1.4. Cytosolic free Calcium Flux Modification/Reactive Oxygen (ROS) Formation/Mitochondria Dependant Apoptosis

Huang *et al.* [49] isolated three new bianthraquinone derivatives, alterporriol K, L, and M, from the endophytic mangrove fungus *Alternaria* sp. ZJ9-6B. Of these three derivatives, alterporriol K and L were moderately active against MDA-MB-435 and MCF-7 human breast cancer cell lines (IC_50_ values of 13.1–29.1 μM) [49]. Moreover, alterporriol L could induce cancer cell apoptosis or necrosis. Furthermore, the reactive oxygen species production, mitochondrial membrane potential, and cytosolic free calcium level were changed after treatment with alterporriol L, suggesting that alterporriol L played vital roles in breast cancer cells, through destroying the mitochondria [196]. The bianthraquinone derivative alterporriol F was previously isolated from the pathogenic fungus *Alternaria porri*. This compound was found to be highly cytotoxic towards HeLa and KB cells, with IC_50_ values of 6.5 and 7.0 μM [197].

Recently, Hu *et al.* [198] reported that rhein could inhibit the purinergic P2X_7_ receptor-mediated rat peritoneal macrophages responses, such as increases in the intracellular cytosolic calcium concentration, pore formation, reactive oxygen species production, attenuation of phagocytosis, and cell apoptosis. P2X_7_ receptor plays important roles in inflammation and immunity. Thus, rhein can be considered as a potential antagonist of the purinergic P2X_7_ receptor, which is a potential therapeutic target for inflammatory diseases.

#### 4.1.5. Transport Inhibition/Synergic Ffects

In 1994, the compound 3,9-dihydroxy-1-methoxy-7-methyl-anthraquinone, identified as the antibiotic C3368-B produced by the fungus strain, *Chrysosporium verrucosum* Tubaki, was found to be a highly-active nucleoside transport inhibitor. This fungal anthraquinone derivative was shown to markedly inhibit thymidine and uridine transport in Ehrlich carcinoma cells, with the half-maximal inhibitory concentration (IC_50_) values of 7.5 and 9.6 μM, respectively. It showed fairly low cytotoxicity towards tumor cells. The IC_50_ values for epidermoid cancer KB cell lines and hepatoma BEL-7402 cells in clonogenic assays were 77 and 69 μM, respectively. At relatively non-cytotoxic concentrations, it markedly enhanced the cytotoxicity of methotrexate, 5-fluorouracil and mitomycin C against KB cells and BEL-7402 cells. It was also found to partly reverse the multi-drug resistance to vincristine and actinomycin D in mouse leukemia L1210/MDR cells. The IC_50_ values were reduced by 4.9-fold (1.75 to 0.36 μM) for vincristine and 3.3-fold (0.39 to 0.12 μM) for actinomycin D. The results suggest that this anthraquinone derivative may be potentially useful in cancer chemotherapy [199].

At present, the role of emodin in combination chemotherapy with standard drugs, in order to reduce toxicity and to enhance efficacy, is pursued vigorously [200]. For example, the synergic effect of arsenic trioxide (currently used to treat acute promyelocytic leukemia), with emodin in combination with clinically achievable doses of docosahexaenoic acid, reduced arsenic concentrations by 100-fold, while still remaining highly toxic to tumor cells [201]. It was also displayed that emodin enhanced the activity of gemcitabine against pancreatic cancer in mice by promoting the mitochondrial-dependent apoptotic pathway. Gemcitabine is currently the standard first-line chemotherapeutic agent for pancreatic cancer [202].

#### 4.1.6. Regulation of Fibrotic and Tumorigenic Mediators

In a way similar to emodin, clinical studies and experiments with animal disease models or different functional cells demonstrated that the anthraquinone rhein (4,5-dihydroxyanthraquinone-2-carboxylic acid) exerted multiple functions including anti-carcinogenesis, antioxidant, anti-inflammation and immunosuppression [198]. For instance, its application notably suppressed the mRNA and protein levels of various fibrotic and tumorigenic mediators, including alpha-smooth muscle actin, type I collagen, fibronectin, N-cadherin, and matrix metalloproteinases in several mammalian cells (rat pancreatic stellate cells, human pancreatic ductal adenocarcinoma cells, and human colon carcinoma cells SW480 and SW620) [203].

#### 4.1.7. Limitation of Vascularization

Recently, the effect of emodin on the growth of transplanted U14 cervical cancer cells in mice and its antitumor mechanism were reported by Zhang *et al.* [204]. Emodin might suppress the growth of cervical cancer by reducing tumor neovascularization, decreasing macrophage′s migration inhibitory factor expression and promoting tumor cell apoptosis. The tumor inhibition rates were 15.83%, 46.92%, and 51.22% in the low-dose emodin group (20 mg/kg), high-dose emodin group (40 mg/kg), and cisplatin group (3 mg/kg), respectively. The tumor inhibition rates were higher in the latter two groups than that in low-dose emodin group. It was also revealed that emodin attenuated tumor cell-induced metastasis and angiogenesis, both *in vitro* and *in vivo* [205]. Finally, emodin has been reported to inhibit the growth of pancreatic cancer PANC-1 cells, which may be related to the demethylation of tumor suppressor genes. The related mechanism may be through the inhibition of methyltransferase expression [206]. Consequently, these results provide important insights into emodin as an anti-invasive agent, for the therapy of human pancreatic cancer at least.

#### 4.1.8. Induction of DNA Damages

Two furofuran precursors of sterigmatocystin, versicolorin A and versicolorin B (*i.e.*, two anthraquinone derivatives), were identified in the culture of the fungus *Aspergillus versicolor* [207]. In a cytotoxicity study of these molecules on human adenocarcinoma lung cells A549, the IC_50_ values in the MTT assay (3-(4,5-dimethyl-2-thiazolyl)2,5-diphenyl-2*H*-tetrazolium bromide cell proliferation assay) were as follows: 109 μM for versicolorin A and 172 μM for versicolorin B. The two compounds were found to exert significant DNA damages, compared to the control in the comet assay. Versicolorin B produced the highest DNA damages [207].

#### 4.1.9. Hydroxy Groups and Hydrogen Bonding between Biomacromolecules

In 2010, Zhang *et al.* [184] reported the isolation of fourteen hydroxyanthraquinone derivatives from the mangrove fungi *Guignardia* sp. No. 4382 and *Halorosellinia* sp. No. 1403. Some of these hydroxyanthraquinones showed potent cytotoxicity to drug-sensitive parental KB and KBv200 cancer cell lines. Among them, one monohydroxyanthraquinone—1-hydroxy-3-methyl-anthraquinone—displayed strong cytotoxicity with IC_50_ values of 3.17 and 3.21 μM to KB and KBv200 cancer cell lines, respectively (MTT assay). The authors suggested that various substituting groups modulated the different contributions of these natural hydroxyanthraquinones towards the anticancer activity. It is believed that hydroxy groups perform important roles by offering hydrogen bonding with biomacromolecules such as proteins. At the same time, the quantity and location of hydroxy groups are fundamental to the activities of the anthraquinone compounds [208]. The compound 1-hydroxy-3-methylanthraquinone, containing only one hydroxy group on position R1, showed the most potent inhibition of growth of KB cells and KBv200 cells. Interestingly, dihydroxyanthraquinones with 1-hydroxy and another hydroxy on another carbon led to the decrease of anticancer activity (*i.e.* 1,8-dihydroxyanthraquinone, 1,8-dihydroxy-3-methylanthraquinone or 1,3-dihydroxy-6-methoxy-8-methylanthraquinone). Hydroxyanthraquinones containing three hydroxy groups at different carbons showed their cytotoxicity IC_50_ values more than 500.0 μM, (*i.e.*, 1,4,7-trihydroxy-2-methoxy-6-methylanthraquinone and 1,3,8-trihydroxy-6-methylanthraquinone). It seems that multi-hydroxy-substitution was unfavorable to the cytotoxic activity . The compound 1-hydroxy-3-methylanthraquinone also induced apoptosis, probably related to mitochondrial dysfunction. Indeed, this compound did not intercalate into DNA according to a DNA binding assay and implied that apoptosis induced by this fungal monohydroxyanthraquinone might not involve DNA intercalation. The apoptosis rates of drug-treated cells with 12.0 μM of this monohydroxyanthraquinone for 48 h were 25.3 and 26.4 for KB and KBv200 cancer cell lines, respectively [184].

#### 4.1.10. Other Compounds

Nemoto *et al.*, in 1996 [209], investigated the antimicrobial and antitumor activities of three benz[a]anthraquinone derivatives, *i.e.*, brasiliquinones A, B, and C (isolated from the pathogenic strain *Nocardia brasiliensis*), against LI210, P388, and drug resistant P388/ADR tumor cells*.* The benz[a]anthraquinone derivatives brasiliquinones B and C were more effective against L1210 tumor cells than brasiliquinone A. The IC_50_ values of the brasiliquinones B and C against L1210 and P388 tumor cells ranged from 2.9 to 7.0 μg/mL. The 3 compounds were active against Gram-positive bacteria including *Mycobacterium* sp.

According to the study by Ge *et al.* [210], three anthraquinone derivatives isolated from the culture of the endophytic fungus *Pleospora* sp. IFB-E006, *i.e.*, altersolanol B, deoxybostrycin, and dactylariol, exhibited significant cytotoxic activity against human colon cancer (SW1116) and leukemia (K562) cell lines, while physcion, 7-methoxy-2-methyl-3,4,5-trihydroxyanthraquinone, and pleospdione (hexahydroanthraquinone) were only weakly or moderately active.

The marine anthraquinone derivative, anhydrofusarubin isolated from the marine endophytic fungus *Fusarium* sp. No. b77, showed a significant inhibition of the growth of HEp2 and HepG2 cells, with IC_50_ values of 8.67 and 3.47 μM, respectively [66,211].

Metabolomic investigations focusing on the marine-derived fungus *Aspergillus* sp. SCSIO F063 have unveiled seven new chlorinated anthraquinones related to averantin, a well-known fungal anthraquinone derivate. One of them, 6-*O*-methyl-7-chloroaverantin, displayed significant inhibition activity against three human tumor cell lines (SF-268, MCF-7, and NCI-H460) with IC_50_ values of 7.11, 6.64, and 7.42 μM, respectively [55].

In a recent review, Wang *et al.* [101] have summarized the sources and structures of 110 natural compounds including marine anthraquinone derivatives and other marine anthracene-9,10-diones mainly extracted from mangrove-derived fungi. They focused on their bioactivities reported between 2008 and mid-2013 and mentioned several moderate to significant anticancer activities.

#### 4.1.11. Carcinogenic Effects

On the other hand, Ueno *et al.* [212] reported that the anthraquinone derivative rugulosin produced by fungi such as *Penicillium rugulosum* exhibited hepatocarcinogenic effects on male mice.

Moreover, the safety and effectiveness of emodin in naturopathic treatment have not been approved by the U.S. Food and Drug Administration (FDA). Side effects of emodin actually include potential carcinogenesis, nausea, diarrhea, and renal failure. Li *et al.* [213] reported that emodin showed no mutagenic activity in the *Salmonella* mutation assay, but caused genotoxicity in the thymidine kinase gene mutation assay in TK6 cells and in the micronucleus test. Results were in accordance with a previous study which indicated that emodin was genotoxic in several assays (comet assay, micronucleus test, and mutation assay in mouse lymphoma L5178Y *tk^+/−^* cells), whereas chrysophanol and physcion (two other anthraquinones often isolated from fungal cultures) showed no effects [214].

Two anthraquinone-type agents—danthron, a drug for constipation, and diacerein, an anti-inflammatory drug for osteoarthritis—were developed and approved by the U.S. FDA. However, danthron was withdrawn by the FDA in 1999 due to the risk of carcinogenesis. Therefore, clinical use of the naturally occurring anthraquinones should be considered cautiously [215].

Finally, all these findings clearly indicate that anthraquinone derivatives from marine and marine-derived fungi might be considered as potent sources of novel anticancer drugs and, at least, promising anti-leukemic agents, anti-invasive agents for human pancreatic and gastric cancers chemotherapy, and antitumor agents for hepatocellular carcinoma, bladder cancer, and others. However, the cytotoxicity caused by quinones is very complex and seems to occur through several mechanisms. Thus, due to differences in structures and characteristics among quinones, and to the dose-dependant responses observed, the molecular mechanism of the toxicity of each compound remains to be fully elucidated.

### 4.2. Special Focus on Protein Kinase Inhibition

The protein kinases are a large family of enzymes that transfer phosphate from adenosine triphosphate (ATP) to proteins as a means of regulating their activity and conformational state. The crucial role of protein kinases in cell signaling, gene expression, and metabolic regulation is highlighted by the fact that nowadays this family of enzymes is the second most important drug target. Actually, abnormal activity of individual protein kinases is often associated with human diseases, especially tumors whose treatment has been so far restricted to cytotoxic and hormonal agents. Many kinase inhibitors are currently in clinical trials, mostly as antitumor drugs, and two of them, Gleevec (STI-571) and rapamycin, are in clinical use for the treatment of a form of leukemia and for the prevention of tissue rejection after organ transplantation, respectively. One major problem with kinase inhibitors is that the human genome encodes 500 different protein kinases; therefore, inhibitors designed to target specifically an individual kinase are likely to bind to closely related kinases as well, thus interfering with other cell functions [216].

Many of the chemical scaffolds or building blocks studied as ATP site-directed kinase inhibitors are based on more or less complex heterocyclic molecules (mainly with nitrogen and oxygen as heteroatoms). The most common scaffolds are derivatives of the following: quinazolines; phenylamino-pyrimidines, pyrido-pyrimidines, pyrrolo-pyrimidines, pyrimido-pyrimidines, or pyrazolo-pyrimidines; pyrrolo-pyridines; indolin-2-ones; purines; pyridinyl-imidazoles or pyrimidinyl-imidazoles; and phthalazines [216].

The interest of the scientific community has been also focused on the anthraquinone family as anthraquinones have been used for the purification of proteins by affinity techniques taking advantage of their nucleotide specific ligand capability. This enables them to interact with ATP, ADP, and NAD binding sites of enzymes such as dehydrogenases, kinases, and ATPases. A potential drawback of these compounds is that their cyclic planar structure confers them the feature of DNA-intercalators with expectable cytotoxic effects. Even with this limit, the optimization of highly specific and selective inhibitors of this category should be exploited [216].

Emodin is a biologically active natural compound extracted from the biomass of many marine or marine-derived fungi that can be chemically classified as an anthraquinone derivative (1,3,8-trihydroxy-6-methylanthraquinone). Several scientific studies have been performed that indicate the vast variety of effects mediated by this compound. Emodin is known to have anti-microbial, immunosuppressive, and anti-inflammatory activities. It exerts anti-proliferative effects in a vast array of cancer cell lines, often enhancing the sensitivity of cancer cells to chemotherapeutic drugs. The efficacy of emodin in inhibiting tumorigenesis is due, at least in part, to its ability to induce apoptosis [217].

Although the exact mechanism(s) of apoptosis induction by emodin remain unclear, several studies have indicated that this compound is an effective inhibitor of protein kinases that are known to regulate a wide range of cellular processes, including apoptosis. Emodin is a cell permeable inhibitor of protein kinase CK2, a constitutively active Ser/Thr kinase that is highly conserved and ubiquitously expressed in eukaryotic cells [217]. CK2 (an acronym derived from the misnomer “casein kinase 2”) is probably the most pleiotropic protein kinase known with more than 300 protein substrates identified. At variance with the great majority of protein kinases which are normally inactive and are turned on only in response to specific stimuli, the enzyme is ubiquitously expressed in all eukaryotic cells. Abnormally high levels of CK2 have been observed in various types of cancer cells as compared to normal tissues, and CK2 is in fact invariably elevated in a wide variety of tumors [218]. Following this pharmaceutical hit with emodin, studies about anthraquinones were expanded with the addition of bromo (brominated anthraquinones exist in some marine fungal biomasses), nitro, amino or bromoacetamido groups, and many compounds were also active (Table 3). Citreorosein (ω-hydroxyemodin) demonstrated similar kinase-inhibiting properties [219], and this kinase inhibiting action of anthraquinones was further linked to an anti-hypertensive potential as therapeutic agents [220]. Thereby, it would be very interesting to investigate the properties of the very large list of natural anthraquinones (over 100 compounds) produced by marine and marine-derived fungi, gathered for the first time in this review (Table 2).

### 4.3. Immunomodulatory Activity

The term “immunomodulation” means the alteration of immune response which may increase or decrease. Enhancement in the immune responsiveness is called immunostimulation and reduction in the immune responsiveness is called immunosuppression. An immunomodulator may be defined as a substance, biological of synthetic, which can stimulate, suppress or modulate any of the components of the immune system including both innate and adaptive arms of the immune response [221]. The essence of immunomodulation is that a pharmacological agent acting under various dose and time regimens displays an immunomodulating effect.

The extreme manifestations of immunomodulating action of biologically active substances are immunosuppression and immunostimulation; hence, both immunostimulating agents and immunosuppressing agents have their own standing, and the search for better agents exerting these activities is becoming a field of major interest all over the world. Natural adjuvants, synthetic agents, and antibody reagents are used as immunosuppressive and immunostimulative agents. However, there are major limitations to the general use of these agents, such as increased risk of infection and generalized effect throughout the immune system. To overcome these problems, a number of drugs from natural source either plant or fungi have been used to alter the human immune system [221], with cyclosporine A, a fungal metabolite, being a successful immunosuppressive agent.

Among anthraquinones from marine-derived and non-marine-derived fungi, carviolin (roseo-purpurin), 1-*O*-methylemodin, and ω-hydroxy-emodin (citreorosein) were found to have moderate immunosuppressive activities. The immunosuppressive activities (IC50 values) of carviolin (roseo-purpurin), 1-*O*-methylemodin, and ω-hydroxyemodin (citreorosein) were calculated against concanavalin A-induced (T cell) and LPS (lipopolysaccharides)-induced (B cell) proliferation of mouse splenic lymphocytes [222].

As immunosuppressive anthraquinones, emodin (1,6,8-trihydroxy-3-methylanthraquinone) [223], aloe-emodin [224], questin (1,6-dihydroxy-8-methoxy-3-methylanthraquinone), and rubrocristin (1,4,6-trihydroxy-8-methoxy-3-methylanthraquinone) were already known [222,225], it was estimated that the immunosuppressive activity (e.g., inhibition of phytohemagglutinin-induced lymphoproliferative responses) of emodin and similar anthraquinones might be partly mediated through H_2_O_2_ generated from its semiquinone form, and the free OH group at the β-position of the anthraquinone nucleus seems to play an important role in its immunosuppressive effect.

### 4.4. Antimicrobial, Antiviral, Antiparasitic Activities

#### 4.4.1. Antimicrobial Activities

The search for components with antimicrobial activity has gained increasing importance in recent times due to growing worldwide concern about the alarming increase in the rate of infection by antibiotic-resistant microorganisms. There has also been a rising interest in research for natural products from marine-microorganisms for the discovery of new antimicrobial agents in the last three decades [2,226]).

The two new hexahydroanthrones tetrahydrobostrycin and 1-deoxytetrahydrobostrycin isolated from the marine-derived fungus *Aspergillus* sp. strain *05F16* collected at a coral reef (Indonesia) demonstrated antibacterial activities. Tetrahydrobostrycin inhibited the growth of *Staphylococcus aureus* and *Escherichia coli*, at 100 mg/disc, with the inhibition zones of 15 and 9.2 mm in diameter, respectively. Another compound, 1-deoxytetrahydrobostrycin from the same strain, was active against *S. aureus* (12 mm at 100 mg/disc). The growth of *Saccharomyces cerevisiae* and *Mucor hiemalis* were not affected by these compounds at 100 mg/disc. It is interesting that the presence of an OH group at C-1 is important for the antibacterial activity of tetrahydrobostrycin against *E. coli*, although the antibacterial activities of these compounds are very weak [54].

Monodictyquinone A (1,8-dihydroxy-2-methoxy-6-methylanthraquinone) was produced from a culture of a marine-derived fungus of the genus *Monodictys*. Its antimicrobial activity was determined against *Bacillus subtilis*, *Escherichia coli*, and *Candida albicans*. This compound showed antibacterial activities against the three species with 2.5 µg/disk [82].

(2*R*,3*S*)-7-ethyl-1,2,3,4-tetrahydro-2,3,8-trihydroxy-6-methoxy-3-methyl-9,10-anthracenedione, a tetrahydroanthraquinone derivative, showed modest antibacterial activity against standard *Staphylococcus aureus* ATCC25923 and methicillin-resistant strains *S. aureus* SK1 with the MIC (Minimum Inhibitory Concentration) values of 128 and 64 µg/mL respectively. The compound was obtained from the EtOAc extract of the culture broth of the mangrove-derived fungus *Phomopsis* sp. PSU-MA214 [92].

Catenarin and 1,4,6,8 tetrahydroxyanthraquinone, products of the *Aspergillus* *glaucus* group exhibited the highest antibacterial activity against exponentialy growing cells of *Bacillus brevis*. The concentration at 1 µg/mL of catenarin and tetrahydroxyanthraquinone resulted in a complete inhibition of the incorporation of uracil and leucine [36].

Trichodermaquinone and Conioanthraquinone were isolated from *T. aureoviride* PSU-F95 and both exhibited antibacterial activity against MRSA (MIC, 200 μg/mL and 8 μg/mL respectively) [88].

Isorhodoptilometrin-1-methyl ether was isolated from *A. versicolor* and exhibited antibacterial activity against the three Gram-positive bacterial strains *Bacillus cereus*, *B. subtilis* and *S. aureus* (inhibition zones 2, 3, and 5 mm at 50 µg/disk, respectively), and the C-6 propanol group is important for its activity, as determined by a comparison with inactive compound 1-methyl emodin [57].

As mentioned in Khamthong *et al.* 2012, anthraquinone-citrinin derivatives are rare natural products. Penicillanthranin A, an anthraquinone-citrinin derivative was isolated from the sea fan-derived fungus *Penicillium citrinium* PSU-F51. This molecule displayed a moderate antimicrobial activity against *Staphylococcus aureus* ATCC25923 with equal MIC values of 16 µg/mL. The same inhibitory effect was observed against *S. aureus* SK1 for penicillanthranin A, which was four fold more active than chrysophanol [88].

Two other natural anthraquinone derivatives, *i.e.* 4-deoxybostrycin and nigrosporin, obtained from the strain *Nigrospora* sp. showed inhibitory effects against mycobacteria. 4-deoxybostrycin also showed significant inhibition of some clinical multidrug-resistant *Mycobacterium* *tuberculosis* strains (maximal inhibitory concentration <15.7 μM) [123].

To date, most of the anthraquinones studied, isolated from various sources (plants, microbes) exhibited more antibacterial than antifungal activities.

The production of a virulence factor, essential for causing SSTIs (skin and soft tissue infections) by *Staphylococcus aureus*, is controlled by quorum sensing (QS), mediated by the accessory gene regulator (agr). From Daly *et al.* [227], ω-hydroxyemodin (OHM), a polyhydroxyanthraquinone isolated from solid-phase cultures of *Penicillium restrictum*, was identified as a suppressor of QS and a compound sought for the further characterization of the mechanism of action in mouse model. At concentrations that are nontoxic to eukaryotic cells and subinhibitory to bacterial growth, OHM prevented agr signaling by all four *S. aureus* agr alleles. OHM inhibited QS by direct binding to AgrA, the response regulator encoded by the agr operon, preventing the interaction of AgrA with the agr P2 promoter. Decreased dermonecrosis with OHM treatment was associated with enhanced bacterial clearance, and reductions in inflammatory cytokine transcription and expression at the site of infection. Furthermore, OHM treatment enhanced the immune cell killing of *S. aureus in vitro* in an agr-dependent manner. These data suggest that bacterial disarmament through the suppression of *S. aureus* QS may bolster the host innate immune response and limit inflammation.

#### 4.4.2. Antiviral Activity

The search for compounds useful in combating viral infections has resulted in relatively few successes. Most of the clinically useful compounds discovered so far have been nucleoside analogues, the usefulness of which has often been limited by the development of toxic side effects and the emergence of drug-resistant viruses. Consequently, the discovery of new non-nucleoside compounds, which are less toxic to host cells and have different mechanisms of action than nucleoside analogues, would be of great value [228].

Some naturally occurring anthraquinones and their derivatives have been studied for antiviral activity against human immunodeficiency virus (HIV) and other retroviruses [229,230,231,232]. Some of these compounds have also been reported to possess *in vitro* antiviral activity against the herpes simplex viruses 1 and 2 (HSV-1, HSV-2), the influenza virus, the vesicular stomatitis virus [233], and the Epstein-Barr virus [234]. Murine Friend leukemia virus infections in mice have also been reported to be inhibited by polycyclic anthraquinone hypericin [235]. However, other investigators have seen no effect of hypericin on this virus infection in mice [232]. Other anthraquinones have been tested against HIV, but very few have been found to be active against this virus [231]. Tang *et al.* [232] have found that some anthraquinones also have virucidal activity against RNA and DNA viruses.

Alterporriol Q and tetrahydroaltersolanol C, isolated from the culture broth and mycelia of *Alternaria* sp. ZJ-2008003, which was collected from a soft coral (*Sarcophyton* sp.*)* from the South China Sea, expressed antiviral activity against the porcine reproductive virus and respiratory syndrome virus, with IC50 values of 39 and 65 μM, respectively [48].

Alizarin, emodin, emodin anthrone, emodin bianthrone, but also other anthraquinones such as quinalizarin, rhein, hypericin, and protohypericin, showed activity against HCMV strain AD-169 (human cytomegalovirus), distinguishable from cytotoxic effects on cells. Of these, quinalizarin had the highest therapeutic index at 3.4, and emodin anthrone and emodin bianthrone the lowest, probably due to their low solubility indices. The EC_50_ values (effective concentrations giving half-maximal response) of alizarin, quinalizarin, and rhein were lower in experiments with the ganciclovir-resistant strain than with the AD-169 virus strain [236].

Aloe-emodin, a bioactive anthraquinone possesses antiviral and anticancer potential [237] reportedly inhibiting replication of varicella-zoster, herpes simplex Types 1 and 2, pseudorabies, influenza, human cytomegalovirus, and/or Japanese encephalitis virus [236,238,239]. Other anthraquinone derivatives like emodin, chrysophanic acid, and hypericin have demonstrated antiviral activity against hepatitis B/C, poliovirus, and HIV [240,241,242]. Anthraquinones directly kill enveloped viruses [238]. Aloe-emodin also inhibits replication of the un-enveloped enterovirus *71 in vitro*, showing Types I and II interferon (IFN) signaling inductions in mammalian cells [239].

Aloe-emodin showed the strongest inhibition of virus yield among emodin and chrysophanol towards MDCK cells. Aloe-emodin treatment caused more than 1-log reduction (equal to 90% effective concentration [EC90]) in virus RNA loads. Subsequent plaque assay determined half maximal inhibitory concentration (IC50) value of aloe-emodin on virus yield. Aloe-emodin showed dose-dependent inhibition of virus-induced cytopathic effect. Infected cells showed about 50% cytopathic effect, those treated with aloe-emodin at concentrations of 1 and 2.5 μg/mL were less than 10% [237].

#### 4.4.3. Antiparasitic Activity

The biological activities of fungal hydroxyanthraquinone derivatives, *i.e.*, 1,3,8-trihydroxy-6-methyl-anthraquinone, aloe-emodin 8-*O*-glucopyranoside, 1,8-dihydroxy-3-methoxy-6-methyl-anthraquinone, and 1,4,5-trihydroxy-7-ethoxy-2-methyl-anthraquinone, isolated from fungal extract of *Drechslera rostrata* and *Eurotium tonpholium* were studied by Awaad *et al.* [243]. Of these fungal anthraquinones, the 1,8-dihydroxy-3-methoxy-6-methyl-anthraquinone from *E. tonpholium* showed a significant anti-leishmanial activity against *Leishmania major* (with an IC_50_ value of 10.38 μg/mL). On the other hand, oral administration of this compound (50 mg/kg) showed very good anti-leishmanial activity.

### 4.5. Other Identified Biological Activities

In a review published in 2015, Chien *et al.* [215] outlined the chemical structure and biological properties of the naturally occurring anthraquinones and their derivatives with an emphasis on recent findings about their therapeutic potential in autoimmune diabetes. So far, 79 naturally occurring anthraquinones have been highlighted, which include emodin, physcion, cascarin, catenarin, and rhein. A large body of literature has demonstrated that the naturally occurring anthraquinones possess a broad spectrum of bioactivities, such as anticancer, antimicrobial, antiinflammatory, but also cathartic, diuretic, vasorelaxing, and phytoestrogenic. This suggests their possible clinical application in many diseases. Despite the advances that have been made in understanding the chemistry and biology of the anthraquinones in recent years, research into their mechanisms of action and therapeutic potential in autoimmune disorders is still at an early stage [244,245,246,247,248,249,250].

#### 4.5.1. Antioxidant Activities

Oxidative stress contributes to free radical-mediated diseases such as aging, atherosclerosis, cancer, ischemic heart disease, diabetes, hyperlipidaemia, hepatotoxicity, and neurodegenerative diseases. There is considerable interest in the isolation of potent radical scavenging compounds from natural resources to treat these pathologies. Natural and synthetic anthraquinones and their derivatives clearly demonstrated their antioxidants potential [63,246,251,252,253,254].

3-*O*-(α-d-Ribofuranosyl)-questin was isolated and identified in *Eurotium rubrum*, an endophytic fungal strain that was isolated from the inner tissue of the stem of the marine mangrove plant *Hibiscus tiliaceus*. The compound was evaluated for its radical scavenging activities by using the 1,1-diphenyl-2-picrylhydrazyl (DPPH) radical scavenging assay and showed weak-moderate activity [63].

From Nemeikaite-Ceniene *et al.* [255] there is a correlation between the cytotoxicity of natural hydroxyanthraquinones (e.g., emodin and chrysophanol) or model quinones on FLK cells (bovine leukemia virus transformed lamb kidney) and HL-60 cells (human promyelocytic leukemia), and the redox cycling reactions inducing the formation of superoxide. This ability can then be determined by the E1 7values (values of single-electron reduction midpoint potential at pH 7.0). They concluded that the rate constants of the single-electron enzymatic reduction of natural hydroxyanthraquinones may serve as a useful tool for the quantitative description of their cytotoxity with the involvement of oxidative stress.

#### 4.5.2. Excretion Functions

##### Diuretic Activity

The diuretic action of emodin and aloe-emodin is probably due to their strong competitive inhibition on Na^+^-K^+^-ATPase activity. From a study of Zhou *et al.* [247], these compounds demonstrated IC_50_ values of 9.8 μg/mL and 19.3 μg/mL and Ki values of 1.33 × 10^−6^ and 7.41 × 10^−6^, respectively.

##### Laxative Activity

Anthranoid laxatives of natural origin, mainly extracted from plants are widely used [256,257]. The basic structure for all anthranoid laxatives is an anthracene ring, to which a hydroxyl or carbonyl function is substitued at C-9, and hydroxy groups at C-8.

Aloe-emodin and chrysophanol are among the most common anthraquinone laxatives [258]. Emodin also forms the basis of a range of purgative anthraquinone derivatives and, from ancient times, has been widely used as a laxative compound**.** It is believed that the presence of hydroxyl groups in position 1 and 8 of the aromatic ring system is essential for the purgative action of the compound [259].

Because of its chemical structure, emodin glycosides (and other anthraquinones) are carried unabsorbed to the large intestine in mammals, where metabolism to the active aglycones takes place by intestinal bacterial flora. The aglycone exerts its laxative effect by damaging epithelial cells, which leads directly and indirectly to changes in absorption, secretion and motility [258,260]. Emodin also inhibits the ion transport (Cl^−^-channels) across colon cells, contributing to the laxative effect [249,261].

There is good evidence that anthrones/anthraquinones, known as active metabolites of emodin-type O- and C-glycosyl compounds, influence the ion transport across colon cells, although the target transport systems have not yet been elucidated. To solve the problem of contradictory explanations about the laxative action of these drugs, a study tested 25 different anthrone/anthraquinone metabolites of plant drugs. Their influence was assayed on different ion transport systems in Ehrlich cells as a model system. Comparing the laxative potency of these substances, with their influence on the different ion transport systems involved in trans-epithelial ion transport, made it possible to exclude some transport processes as primary targets of the drugs. The results showed that Na^+^-K^+^-2Cl- cotransport was not inhibited by any of the substances tested and that Na^+^/K^+^-ATPase (pump) was inhibited by those 1,8-dihydroxyanthrones/anthraquinones that bear an additional phenolic hydroxyl group. This inhibition is indirect by interference with oxidative ATP production. However, there is no direct correlation to laxative action. In addition, cation channels were not influenced by these drugs, and Cl^−^-channels were inhibited significantly by those drugs that also showed a laxative action. These results make it very likely that inhibition of Cl**^−^**-channels is the primary action responsible for the laxative action. Interference with oxidative ATP production as an additional effect may explain the known synergistic action described for the combination of different anthrones/anthraquinones or anthranoid drugs, respectively [261].

In addition, 1,3,6,8-trihydroxymethylanthraquinone was used in a patented laxative preparation for intravascular injection, active by stimulating the neuromuscular junction of the bowel wall [262].

Studies in humans have also suggested tumor promoting activities for these laxatives. Although the short-term use of these substances is generally safe, long-term utilization cannot be recommended.

#### 4.5.3. Vasorelaxant or Contractile Effects

In a first study of Huang *et al.* [263], the vasorelaxant effect of emodin was assayed as the ability to relax rat thoracic aortic rings precontracted with phenylephrine. The vasorelaxant activity of emodin was expressed as a percentage of relaxation of the maximal tension increase produced by phenylephrine. The concentration evoking 50% relaxation (IC_50_ value) showed that emodin exhibits vasorelaxant effect, and dose-dependently relaxed the contractile responses of rat aortic rings. In the same study, emodin also dose-dependently suppressed the responses of human mononuclear cells to phytohemagglutininand mixed lymphocytereaction. Thus, this compound may be useful as a new template for the development of better immunosuppressive agents, with vasorelaxant actions useful against transplantation rejection and autoimmune disease.

The possible mechanism underlying the vasorelaxant effect of emodin was investigated in a second study by Huang *et al.* [264]. Emodin dose-dependently relaxed isolated vascular rings of several vessels in animals and human, in case of induced contraction. The study also investigated the inhibition, attenuation, or potentiation of relaxation response to emodin by various compounds. It suggested that the vasorelaxant effect of emodin might be mainly due to cGMP accumulation, as a result of guanylate cyclase activation by free radicals and/or hydrogen peroxide generated from semiquinone.

The effects of emodin on skeletal muscle were studied in a mouse-isolated diaphragm and in sarcoplasmic reticulum (SR) membrane vesicles [265]. Emodin dose-dependently caused muscle contracture, simultaneously depressing twitch amplitude. Neither tubocurarine nor tetrodotoxin (neuromuscular non-depolarizing agents) blocked the contraction, suggesting that it was caused myogenically. These data suggest that muscle contraction induced by emodin is dose-dependent and is due to Ca^2+^ release from the SR of skeletal muscle, as a result of oxidation of the ryanodine receptor and influx of extracellular Ca^2+^ through voltage-dependent Ca^2+^ channels of the plasma membrane.

#### 4.5.4. Effects on Lipid and Glucose Metabolism

Several drawbacks of many pharmaceutic drugs used for the treatment of Diabetes mellitus have contributed to the use of “natural” products. Emodin, which was extensively studied, has anti-inflammatory, analgesic [266], and antipancreatitic [267] effects. Its anti-diabetic activity was investigated by evaluating its hypoglycaemic and hypolipidaemic effects, together with its potential effects on L-type calcium-channels in dyslipidaemic-diabetic rats. The results demonstrated significant dose-dependent reductions in blood glucose, serum total cholesterol, triglycerides, free fatty acids, and malonaldehyde in dyslipidaemic-diabetic rats. In addition, emodin caused dose-dependent increases in their plasma superoxide dismutase activity. The results suggest that emodin has antidiabetic and lipid-modulating effects that involve, in part, upregulation of L-type calcium channel expression in the pancreas and heart of dyslipidaemic-diabetic rats [268].

The antidyslipidemic effect of several anthraquinone derivatives as chrysophanol and emodin obtained from *Rheum emodi*′s rhizomes was evaluated. These compounds significantly reduced plasma lipid levels. The most active compound emodin showed significant lipid-lowering activity in the HFD-fed model. The effect of emodin on enzymes modulating lipid metabolism confirms and supports its efficiency as a potent antidyslipidemic agent [269].

#### 4.5.5. Estrogenic Activity

Insufficiency of endogenous estrogen secretion is known to cause several physical disorders in postmenopausal women, such as osteoporosis, hypercholesteremia, and symptoms of menopause. Synthetic estrogen-replacement therapy has been reported to be effective for these diseases. Recently, the estroenic activity of phytoestrogens was reported. A study of these compounds, which are distributed in vegetables, fruits, and medicinal plants, evaluated the estrogenic activity of the methanolic extracts from several medicinal herbs. The study was guided by the detection of a proliferative activity of MCF-7,1 (estrogen-sensitive cell line) [244]. A bioassay-guided separation led to emodin, which enhanced proliferation of MCF-7 from 1 to 10 mM in a concentration-dependent manner. Aloe-emodin and chrysophanol showed weak activity. By comparison of the activities, the 6-hydroxyl group seemed to be essential for enhancement of the estrogenic activity.

To clarify the affinity of the active anthraquinones to human estrogen receptors ERα and Erβ, a competitive binding assay was performed using 17β-estradiol. Aloe-emodin and chrysophanol did not compete with ERα and ERβ for 17β-estradiol binding at a high concentration (40mM). Conversely, emodin competed for 17β-estradiol binding with both ERα and ERβ.

Concerning the structure–activity relationships of anthraquinones regarding the oestrogenic activity, it is quite clear that the unchelated hydroxyl group is essential for a strong estrogenic competency. This is the first report for estrogenic activity of anthraquinones. The findings that emodin bonds with human ERα and ERβ may be useful for replacement therapy for human menoxenia and post-menopausal diseases.

## 5. Conclusions

The recent developments in technology helped in promoting the increased use of natural molecules in industries. Thereby, the microorganisms naturally producing bioactive or useful extrolites recently appeared as a great source of potentially interesting molecules. Opposite to plants and insects, these easily renewable and upscalable resources (short production cycles), independent of seasonality, can give regular productions with potentially higher yields. Unfortunately, far more than the chemical synthetic routes, the biological synthesis requires a deep comprehension of the phenomena involved, and often implies very thin adjustments. This is one of the reasons why some have expressed doubts about the successful production and commercialization of fermentation-derived compounds. Others dealt with the high capital investment requirements for fermentation facilities and the extensive and lengthy toxicity studies required by regulatory agencies. However, several microbial genera are capable of producing highly original compounds in large quantities, and could meet the economic needs of companies, if we are ready to embark on the challenges of the production of natural molecules through biotechnology. For now, the question is: Can the recent and future research efforts on the fungal strains, the productions′ methods, and the biosynthetic improvments reveal secure ways to produce targeted molecules? Thus, it seems that anthraquinoid compounds produced by fungi are quite new tracks for research and applications.

## Figures and Tables

**Table 1:** Marine-derived fungi producing anthraquinones and some derivatives.

Genus	Species/Strain No	Name of Compounds Produced	Source of Isolation	Refs.
***Alternaria***	*Al. eichorniae*	4-deoxyBostrycin, Bostrycin	Mar. Plant pathogen	[46]
	*Al*. (SK11)	(+) α S-alterporriol C, 6-methylquinizarin, Alterporriol S, Austrocortinin	Mangrove Plant end.	[47]
	*Al*. sp. ZJ-2008003	Alterporriol C, K–R, Altersolanol B and C, Macrosporin	Mar. Org end.	[48]
	*Al*. sp. ZJ9-6B	Alterporriols C–M, Altersolanol A, Dactylariol, Macrosporin, Physcion, TetrahydroAltersolanol B	Mar. Plant end.	[49]
***Aspergillus***	*A. glaucus*	10,10′-dimer of Emodin and Physcion, Catenarin, Cynodontin, Emodin, Erythroglaucin, Helminthosporin, Physcion, Questin, Rubrocristin, Tritisporin, Variecolorquinone A	Mangrove sed.	[50,51,52,53]
	*A*. sp. 05F16	1-deoxytetrahydrobostrycin, Tetrahydrobostrycin	Algal end.	[54]
	*A*. sp. SCSIOF063	(1′*S*)-7-chloroaverantin, 1′-*O*-methylaverantin 1′-*O*-methyl-7-chloroaverantin, 6-*O*-methyl-7-chloroaverantin, 6-*O*-methyl-7-chloroaverythrin, 6-*O*-methyl-7-bromoaverantin, 6,1′-*O*,*O*-dimethyl-7-chloroaverantin, 6,1′-*O*,*O*-dimethyl-7-bromoaverantin, 6,1′-*O*,*O*-dimethylaverantin, 7-chloroaverantin-1′-butyl ether, 7-chloroaverythrin	Sed.	[55]
	*A. variecolor* B-17	(2*S*)-2,3-dihydroxypropyl1,6,8-trihydroxy-3-methyl-9,10-dioxoanthracene-2carboxylate, Catenarin, Emodin, Fallacinol, Physcion, Erythroglaucin, Questin, Questinol, Rubrocristin, Variecolorquinone A,	Sed.	[56]
	*A. versicolor*	7-hydroxyemodin 6,8-methyl ether, Emodin, Isorhodoptilometrin-methyl ether, Methyl emodin	Algal end.	[57]
	*A. versicolor* EN-7 (Genbank no EU042148)	6,8-di-*O*-methylversiconol 6,8-di-*O*-methylnidurufin 6,8-di-*O*-methylaverantin 6,8-di-*O*-methylversicolorin A, Aversin: (−)-isomer	Algal end.	[58]
***Curvularia***	C. *lunata*	Cytoskyrin A, Lunatin	Mar. Org end.	[50,51,59,60]
***Eurotium***	*E. cristatum (ECE)*	Catenarin, Emodin, Erythroglaucyn, Physcion, Physcion anthrone, Questin, Rubrocristin	Mar. Org end.	[36,61]
	*E. repens*	Catenarin, Erythroglaucyn, Physcion, Physcion anthrone	Mar. Org end.	[36,62]
	*E. rubrum*	6,3-*O*-(α-d-ribofuranosyl)-questin, Questin	Mar. Plant end.	[63]
***Unidentified***	Fungus Isolate 1850 and 2526	Averufin, Nidurufin, versicolorin C	Mar. Plant end.	[64]
	Fungus ZSUH-36	1′-*O*-methyl averantin, 6,8-di-*O*-methyl averufanin, 6,8-di-*O*-methyl averufin, 6,8,1′-tri-*O*-methyl averantin, Versicolorin C	Mar. Plant end.	[65]
***Fusarium***	*F*. sp. No. B77	5-acetyl-2-methoxy-1,4,6-trihydroxy-anthraquinone	Mangrove Plant end.	[66]
	*F*. sp. ZZF60	6,8-dimethoxy-1-methyl-2-(3-oxobutyl) anthraquinone	Mangrove Plant end.	[67]
	*F*. sp. No. ZH-210	Fusaquinon A,B,C	Mangrove sed.	[68]
	*F*. sp. PSU-F14, *F*. sp. PSU-F135	Austrocortirubin, Bostrycin	Mar. Org end.	[69]
***Halorosellinia***	*H*. sp. (No. 1403)	1,4,5,6,7,9-hexahydroxy-2-methoxy-7-methyl-5β,9β, 8aβ,6α,10aα—hexahydroanthracene10(10aH)-one, Austrocortirubin, Demethoxyaustrocortirubin, Hydroxy-9,10-anthraquinone, SZ-685C	Mar. Plant-derived	[70,71]
***Lichens***	*Arthonia elegans, Biatorella conspersa, B. ochrophora, Pyrenula cerina, Sphaerophorus fragilis, Stereocaulon corticatulum,v. procerum, Trypethelium aeneum, T. aureomaculata, etc.*	Physcion	Lichens	[72]
	*Caloplaca* sp.	Phallacinol (=Teloschistin=Fallacinol))	Lichen	[73,74]
	*Caloplaca ehrenbergii*, *C. schaereri*, *C. spitsbergensis*, *etc*.	1-*O*-methyl-7-chloroemodin, 7-chloro-1,6,8-trihydroxy-3-methyl-10-anthrone, 7-chlorocitreorosein, 7-chloroemodic acid, 7-chloroemodin, 7-chloroemodinal, Emodin, Phallacinol, Physcion	Lichens	[75,76,77]
	*Gliocladium* sp. T 31	Citreorosein, Emodin, Isorhodoptilometrin	Lichen	[78]
	*Letrouitia hafellneri, L. leprolytoides*	7-chloroemodinal, 7-chloroemodin, Fragilin, Physcion	Lichens	[79,80]
***Microsphaeropsis***	*M*. sp.	1,3,6,8-tetrahydroxyanthraquinone, 1,3,6,8-tetrahydroxy-2-(1-hydroxyethyl)anthraquinone 1,3,6,8-tetrahydroxy-2-(1-methoxyethyl)anthraquinone 1,2,3,6,8-pentahydroxy-7-(1-methoxyethyl)anthraquinone	Mar. Org end.	[81]
***Monodictys ***	*M*. sp.	Chrysophanol, Emodin, Monodictyquinone A, Pachybasin	Mar. Org end.	[14,82]
***Nigrospora***	*N*. spp.	1-deoxytetrahydrobostrycin, 4-deoxybostrycin, Bostrycin, 4a-*epi*-9α-methoxydihydrodeoxybostrycin, 10-deoxybostrycin	Mar. Plant/Org end.	[68,83,84]
	*N*. sp. MA75	4-deoxybostrycin, Bostrycin	Marine	[85]
	*N*. sp. 1403	4-deoxybostrycin, Bostrycin	Mangrove	[86]
***Paecilomyces***	*P*. sp. (Tree 1-7)	Chrysophanol, Emodin	Mangrove	[87]
***Penicillium***	*P. citrinum* PSU-F51 (Accession no JQ66600)	Chrysophanol, Citreorosein, Emodin, Penicillanthranins A and B	Mar. Org end.	[88]
	*P. chrysogenum*	Skyrin	Salt lake	[89]
	*P. flavidorsum* SHK1-27	6,8-*O*-dimethylaverufin, 8-*O*-methylaverufin, Averufin, Averantin, Versiconol, Versicolorin A&B, Nidurufin	Marine	[90]
	*P. oxalicum* 2-HL-M-6	Aloe emodin, Chrysophanol, Citreorosein, Citreorosein-3-*O*-sulfate, Emodin, Emodin-3-*O*-sulfate, Isorhodoptilometrin	Mangrove sed.	[91]
***Phomopsis***	*P*. sp. PSU-MA214	Phomopsanthraquinone, 1-hydroxy-3-methoxy-6-methylanthraquinone, Ampelanol, Macrosporin	Mangrove Plant endo	[92]
***Stemphylium***	*S*. sp. 33231	2-*O*-acetylaltersolanol B, Alterporriol T–W, Altersolanol B&C, Auxarthrol C, Macrosporin	Mangrove Plant end.	[93]
	*S. globuliferum*	6-*O*-methylalaternin, Acetylalterporriol D and E, Alterporriol D and E, Altersolanol A,B and C, Dihydroaltersolanol B and C, Macrosporin, Stemphylanthranol A and B	Salt lake Plant end.	[94]
***Trichoderma***	*T. aureoviride* PSU-F95	Coniothranthraquinone 1, Trichodermaquinone	Mar. Org end.	[88]
***Xylaria***	*X*. sp. 2508	Altersolanol A, Bostrycin, Deoxybostrycin, Xylanthraquinone	Marine	[95]

Abreviations*:* Mar. Plant end.: marine plant endophyte; Mar. Org. end.: marine organism endophyte other than plant; Sed.: sediment.

**Table 2:** Structural diversity of anthraquinoid compounds identified in marine-derived fungi (increasing mol. mass.).

Mol. Formula/*Mol. mass*	Trivial name	Structure	IUPAC NAME	Source	Refs
C_14_H_8_O_3_/*224*	Hydroxy-9,10-anthraquinone 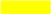	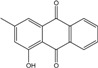	1-hydroxy-3-methylanthraquinone	*Halorosellinia* sp. No. 1403	[70]
C_15_H_10_O_3_/*238*	Pachybasin 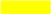	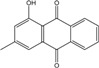	1-hydroxy-3-methylanthraquinone	*Monodictys* sp.	[14,82]
C_15_H_10_O_4_/*254*	Chrysophanol 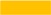	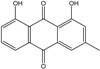	1,8-dihydroxy-3-methylanthraquinone	*Monodictys* sp. *Paecilomyces* sp. *P. citrinum* PSU-F51 *P. oxalicum* 2-HL-M-6	[14,82] [87] [88] [91]
C_15_H_10_O_4_/*254*	6-Methylquinizarin	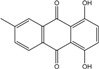	1,4-dihydroxy-methylanthraquinone	*Al.* sp. (SK11)	[47]
C_16_H_12_O_4_/*268*	1-Hydroxy-3-methoxy-6-methylanthraquinone	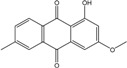	----	*Phomopsis* sp. PSU-MA214	[92]
C_15_H_10_O_5_/*270*	Aloe emodin 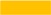	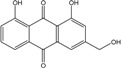	1,8-dihydroxy-3-(hydroxymethyl)anthraquinone	*P. oxalicum* 2-HL-M-6	[91]
C_15_H_10_O_5_/*270*	Emodin 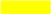	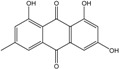	1,3,8-trihydroxy-6-methylanthraquinone	*A. glaucus A. variecolor* B-17 *A. versicolor Caloplaca* spp. *(*e.g., *C. ehrenbergii, C. schaereri, C. spitsbergensis, etc.) Eurotium Cristatum Gliocladium* sp*.* T31 *Monodictys* sp. *Paecilomyces* sp. *P. citrinum* PSU-F51 *P. oxalicum* 2-HL-M-6	[91] [56] [61] [75,76,77,79,96] [36] [78] [14,82] [87] [88] [57]
C_15_H_10_O_5_/*270*	Helminthosporin 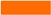	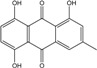	1,5,8-trihydroxy-3-methylanthraquinone	*A. glaucus*	[50,51,52,53]
C_14_H_8_O_6_/*272*	1,3,6,8-Tetrahydroxyanthraquinone 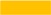	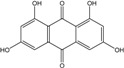	----	*A.* *versicolor Microsphaeropsis*	[97] [81]
C_15_H_14_O_5_/*274*	Coniothranthraquinone 1	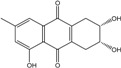	(2*S*,3*R*)-2,3,5-trihydroxy-7-methyl-1,2,3,4-tetrahydroanthraquinone	*Trichoderma aureoviride* (PSU-F95)	[88,98]
C_16_H_12_O_5_/*284*	1-Methylemodin 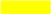	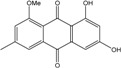	1,3-dihydroxy-8-methoxy-6-methylanthraquinone	*A. versicolor*	[57]
C_16_H_12_O_5_/*284*	Austrocortinin 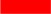	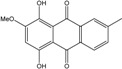	1,4-dihydroxy-2-methoxy-7-methylanthraquinone	*Al.* sp. (SK11)	[47]
C_16_H_12_O_5_/*284*	Macrosporin 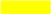	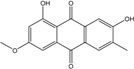	1,7-dihydroxy-3-methoxy-6-methylanthraquinone	*Al.* sp. ZJ9-6B *Al.* sp. ZJ-2008003 *Phomopsis* sp. PSU-MA214 *Stemphylium globuliferum Stemphylium* sp. 33231	[49] [48] [92] [94] [93]
C_16_H_12_O_5_/*284*	Marcrospin	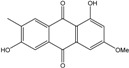	1,6-dihydroxy-3-methoxy-7-methylanthraquinone	*Al.* sp. ZJ9-6B	[49]
C_16_H_12_O_5_/*284*	Monodictyquinone A 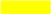	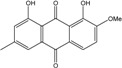	1,8-dihydroxy-2-methoxy-6-methylanthraquinone	*Monodictys* sp.	[14,82]
C_16_H_12_O_5_/*284*	Phallacinol/Fallacinol 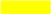	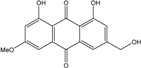	1,8-dihydroxy-3-(hydroxy-methyl)-6-methoxyanthraquinone	*A. variecolor* B-17 *Caloplaca* spp. *(*e.g., *C. ehrenbergii, C. schaereri, C. spitsbergensis, etc.)*	[99] [73,74,76,77,79,96]
C_16_H_12_O_5_/*284*	Physcion 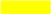	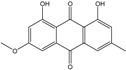	1,8-dihydroxy-3-methoxy-6-methylanthraquinone	*Al.* sp. ZJ9-6B *A. glaucus A. variecolor* B-17 *Eurotium repens Eurotium cristatum Caloplaca* spp. *(*e.g., *C. ehrenbergii, C. schaereri, C. spitsbergensis, etc.) Letrouitia hafellneri*, *L. leprolytoides*, *Arthonia elegans*, *Biatorella conspersa*, *B. ochrophora*, *Pyrenula cerina*, *Sphaerophorus fragilis*, *Stereocaulon corticatulum*, *v. procerum*, *Trypethelium aeneum*, *T. aureomaculata*	[49] [50,51,52,53] [56] [62] [36,61] [75,76,77,79,96] [72,80]
C_16_H_12_O_5_/*284*	Questin 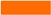	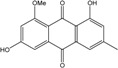	1,6-dihydroxy-8-methoxy-3-methylanthraquinone	*A. glaucus A. variecolor* B-17 *Eurotium cristatum (ECE) Eurotium rubrum*	[50],[51] [56] [52,53] [36,61,63]
C_15_H_10_O_6_/*286*	Catenarin 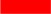	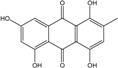	1,4,5,7-tetrahydroxy-2-methylanthraquinone	*A. glaucus A. variecolor* B-17 *Eurotium cristatum (ECE) Eurotium repens*	[50,51] [56] [52,53] [36,62]
C_15_H_10_O_6_/*286*	Citreorosein 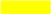	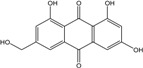	ω-hydroxyemodin (OHM) or 1,3,8-trihydroxy-6-(hydroxymethyl) anthraquinone	*Gliocladium*. sp. T31 *P. citrinum* PSU-F51 *P. oxalicum* 2-HL-M-6	[78] [88] [91]
C_15_H_10_O_6_/*286*	Cynodontin 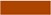	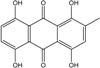	1,4,5,8-tetrahydroxy-2-methylanthraquinone	*A. glaucus*	[50,51,52,53]
C_15_H_10_O_6_/*286*	Lunatin	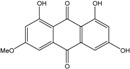	1,3,8-trihydroxy-6-methoxyanthraquinone	*Curvularia lunata*	[50,51,59,60]
C_15_H_10_O_7_/*286*	Tritisporin 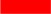	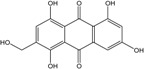	1,4,5,7-tetrahydroxy-2-(hydroxylmethyl) anthraquinone	*A. glaucus*	[50,51,52,53]
C_15_H_14_O_6_/*290*	Trichodermaquinone	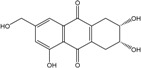	(2*S*,3*R*)-2,3,5-trihydroxy-7-(hydroxylmethyl)-1,2,3,4-tetrahydroanthraquinone	*Trichoderma aureoviride* (PSU-F95)	[88,98]
C_15_H_14_O_6_/*290*	Demethoxyaustrocortirubin 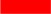	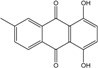	1,4-dihydroxy-6-methylanthraquinone	*Halorosellinia* sp. No. 1403	[70,71]
C_15_H_14_O_6_/*290*	7-Hydroxyemodin 6,8-methyl ether	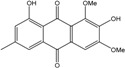	2,8-dihydroxy-1,3-dimethoxy-6-methyl anthraquinone	*A. versicolor*	[57]
C_16_H_10_O_4_/*300*	Erythroglaucin 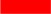	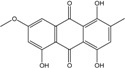	1, 4, 5-trihydroxy-7-methoxy-2-methylanthraquinone	*A. glaucus A. variecolor* B-17 *Eurotium cristatum (ECE) Eurotium repens*	[50,51,52,53] [56] [36,61] [36]
C_16_H_12_O_6_/*300*	Carviolin	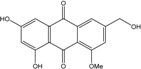	1,3-dihydroxy-6-(hydroxymethyl)-8-methoxyanthraquinone	*P. dravuni*	[100]
C_16_H_12_O_6_/*300*	Questinol	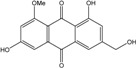	1,6-dihydroxy-3-(hydroxymethyl)-8-methoxyanthraquinone	*A. variecolor* B-17	[56]
C_16_H_12_O_6_/*300*	Rubrocristin 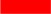	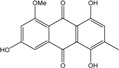	1,4,7-trihydroxy-5-methoxy-2-methylanthraquinone	*A. glaucus A. variecolor* B-17 *Eurotium cristatum (ECE)*	[50,51,52,53] [56] [36,61]
C_16_H_12_O_6_/*300*	6-*O*-methylalaternin 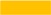	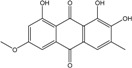	1,2,8-trihydroxy-6-methoxy-3-methylanthraquinone	*Stemphylium globuliferum*	[94]
C_16_H_12_O_6_/*300*	1,4,6-Trihydroxy-2-methoxy-7-methyl-anthraquinone 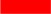	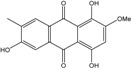	3,5,8-trihydroxy-7-methoxy-2-methylanthraquinone	*Halorosellinia* sp. No. 1403	[70]
C_15_H_9_O_5_Cl/*304*	7-Chloroemodin	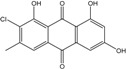	----	*Caloplaca* spp. *(*e.g.*, C. ehrenbergii, C. schaereri, C. spitsbergensis, etc.*)	[75,76,77,79,96]
C_16_H_16_O_6_/*304*	Altersolanol B 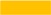	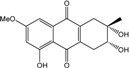	(2*S*,3*R*)-2,3,5-trihydroxy-7-methoxy-2-methyl-1,2,3,4-tetrahydroanthraquinone	*Al.* sp. ZJ-2008003 *Stemphylium* sp. 33231	[48] [93]
C_16_H_18_O_6_/*306*	Fusaquinon A	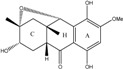	(2*R*,3*S*,4a*R*,9*S*,9a*S*)-3,5,8-trihydroxy-7-methoxy-2-methyl-2,3,4,4a,9,9a-hexahydro-2,9-epoxyanthracen-10(1*H*)-one	*Fusarium* sp. No. ZH-210	[68]
C_16_H_18_O_6_/*307*	Dihydroaltersolanol B 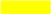	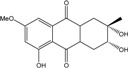	(2*S*,3*R*)-2,3,5-trihydroxy-7-methoxy-2-methyl-1,2,3,4,4a,9a-hexahydroanthraquinone	*Stemphylium globuliferum*	[94]
C_15_H_19_O_7_/*311*	Xylanthraquinone	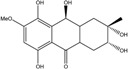	----	*Xylaria* sp. 2508	[95]
C_17_H_14_ O_6_/*314*	Isorhodoptilometrin 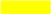	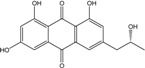	(*R*)-1,3,8-trihydroxy-6-(2-hydroxypropyl)anthraquinone	*Gliocladium* sp. T31 *P. oxalicum* 2-HL-M-6	[78] [91]
C_16_H_12_O_7_/*317*	1,3,6,8-Tetrahydroxy-2-(1-hydroxyethyl) anthraquinone 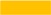	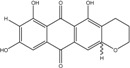	1,3,6,8-tetrahydroxy-2-(1-hydroxyethyl) anthracene-9,10-dione	*Microsphaeropsis*	[81]
C_16_H_11_O_5_Cl/*318*	1-*O*-Methyl-7-chloroemodin	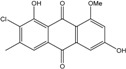	2-chloro-1,6-dihydroxy-8-methoxy-3-methylanthraquinone	*Caloplaca* spp. *(*e.g., *C. ehrenbergii, C. schaereri, C. spitsbergensis, etc.)*	[75,76,77,79,96]
C_15_H_9_O_6_Cl/*320*	7-Chlorocitreorosein	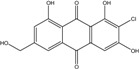	----	*Caloplaca* spp. *(*e.g., *C. ehrenbergii, C. schaereri, C. spitsbergensis, etc.)*	[75,76,77,79,96]
C_16_H_16_O_7_/*320*	Austrocortirubin 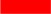	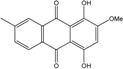	1,4-dihydroxy-2-methoxy-7-methylanthraquinone	*Fusarium* spp. PSU-F14 and PSU-F135 *Halorosellinia* sp. No. 1403 *Nigrospora* sp. ZJ-2010006	[69] [70,71] [84]
C_16_H_16_O_7_/*320*	Altersolanol C 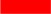	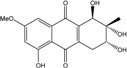	(1*R*,2*R*,3*R*)-1,2,3,5-tetra-hydroxy-7-methoxy-2-methyl-1,2,3,4-tetra-hydroanthraquinone	*Al.* sp. ZJ9-6B *Al.* sp. ZJ-2008003 *Stemphylium* sp. 33231	[49] [48] [93]
C_16_H_16_O_7_/*320*	4-Deoxybostrycin 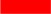	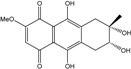	(2*R*,3*S*,4a*S*,9a*S*,10*R*)-2,3,5,8,10-pentahydroxy-6-methoxy-3-methyl-1,3,4,4a,9a,10-hexahydroanthracen-9(2*H*)-one	*Nigrospora* sp. 1403 *Nigrospora* sp. MA75	[68,83,84,86,101] [85]
C_16_H_16_O_7_/*320*	2,3,5,8-Tetrahydroxy-7-methoxy-2-methyl-1,2,3,4-tetrahydroanthraquinone	----	2,3,5,8-tetrahydroxy-7-methoxy-2-methyl-1,2,3,4-tetrahydroanthracene-9,10-dione	*Al. eichorniae*	[46]
C_16_H_16_O_7_/*321*	10-Deoxybostrycin 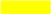	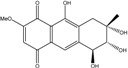	---	*Nigrospora* sp.	[84]
C_16_H_18_O_7_/*323*	Dihydroaltersolanol C 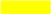	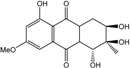	(1*R*,2*R*,3*R*)-1,2,3,5-tetra-hydroxy-7-methoxy-2-methyl-1,2,3,4,4a,9a-hexahydroanthraquinone	*Stemphylium globuliferum*	[94]
C_16_H_20_O_7_/*324*	Fusaquinon C 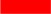	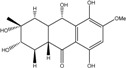	(2*S*,3*R*,4a*R*,9a*R*,10*S*)-2,3,5,8,10-pentahydroxy-6-methoxy-3-methyl-1,3,4,4a,9a,10-hexahydroanthracen-9(2*H*)-one	*Fusarium* sp. No*.* ZH-210	[68]
C_16_H_21_O_7_/*325*	1-Deoxytetrahydrobostrycin 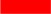	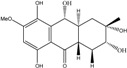	(2*R*,3*S*)-2,3,5,8,10-pentahydroxy-6-methoxy-3-methyl-1,3,4,4a,9a,10-hexahydroanthracen-9(2*H*)-one	*A.* sp. *05F16 Nigrospora* sp*.*	[54] [83]
C_15_H_20_O_8_/*328*	Fragilin 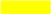	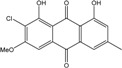	2-chloro-1,8-dihydroxy-3-methoxy-6-methylanthraquinone	*Letrouitia hafellneri L. leprolytoides*	[79,80]
C_17_H_12_O_7_/*328*	5-Acetyl-2-methoxy-1,4,6-trihydroxyanthraquinone	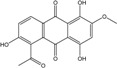	----	*Fuarium* sp. B77	[66]
C_18_H_16_O_6_/*328*	Isorhodoptilometrin-1-methylether	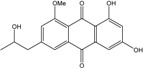	1,3-dihydroxy-6-2-hydroxypropyl-8-methoxyanthraquinone	*A. versicolor*	[57]
C_17_H_14_O_7_/*329*	1,3,6,8-Tetrahydroxy-2-(1-methoxyethyl)anthraquinone 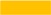	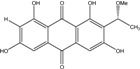	---	*Microsphaeropsis*	[81]
C_18_H_20_O_6_/*332*	Phomopsanthraquinone	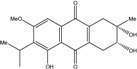	(2*R*,3*S*)-7-ethyl-1,2,3,4-tetrahydro-2,3,8-trihydroxy-6-methoxy-3-methylanthraquinone	*Phomopsis* sp. PSU-MA214	[92]
C_16_H_16_O_8_/*336*	Altersolanol A	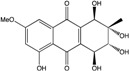	(1*R*,2*S*,3*R*,4*S*)-1,2,3,4,5-pentahydroxy-7-methoxy-2-methyl-1,2,3,4-tetrahydroanthraquinone	*Stemphylium globuliferum Xylaria* sp. *2508*	[94] [95]
C_16_H_16_O_8_/*336*	Bostrycin	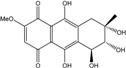	(5*S*,6*R*,7*S*)-5,6,7,9,10-pentahydroxy-2-methoxy-7-methyl-5,6,7,8-tetrahydroanthracene-1,4-dione	*A.* sp. *strain* 05F16 *Al. eichorniae Fusarium* spp. PSU-F14/PSU-F135 *Halorosellinia* sp. No. 1403 *Nigrospora* sp. *Xylaria* sp. 2508	[46,102] [69] [68,86,101,103] [84] [54] [95]
C_16_H_18_O_8_/*338*	SZ-685C	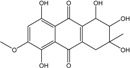	1,2,3,5,8-pentahydroxy-6-methoxy-3-methyl-1,2,3,4-tetrahydroanthraquinone	*Halorosellinia* sp. No. 1403	[70,101,104,105,106,107]
C_16_H_20_O_8_/*340*	Fusaquinon B 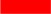	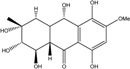	(1*R*,2*S*,3*R*,4a*R*,9a*S*,10*S)*-1,2,3,5,8,10-hexahydroxy-6-methoxy-3-methyl-1,3,4,4a,9a,10-hexahydroanthracen-9(2*H*)-one	*Fusarium* sp. No. ZH-210	[68]
C_16_H_21_O_8_/*340*	Tetrahydroxybostrycin	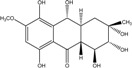	1,2,3,5,8,10-hexahydroxy-6-methoxy-3-methyl-1,3,4,4a,9a,10-hexahydroanthracen-9(2*H*)-one	*A.* sp*.* 05F16 *Nigrospora* sp. MA75	[54] [85]
C_18_H_12_O_7_/*340*	Versicolorin C	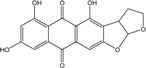	4,6,8-trihydroxy-3,3a-dihydroanthra[2,3-b]furo[3,2-d]furan-5,10(2*H*,12a*H*)-dione	Fungus ZSUH-36 Fungus Isolate 1850 and isolate 2526	[65] [64]
C_18_H_18_O_7_/*346*	2-*O*-Acetylaltersolanol B 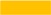	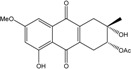	(2*R*,3*S*)-3,8-dihydroxy-6-methoxy-3-methyl-9,10-dioxo-1,2,3,4,9,10-hexahydroanthracen-2-yl acetate	*Stemphylium* sp. 33231	[93]
C_17_H_14_O_8_/*347*	1,2,3,6,8-Pentahydroxy-7-(1-methoxyethyl)anthraquinone 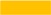	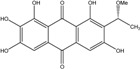	1,2,3,6,8-pentahydroxy-7-(1-methoxyethyl)anthracene-9,10-dione	*Microsphaeropsis*	[81]
C_15_H_10_O_8_S/*350*	Emodin-3-*O* Sulfate 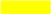	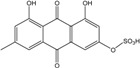	4,5-dihydroxy-7-methyl-9,10-dioxo-9,10-dihydroanthracen-2-yl hydrogen sulfate	*P. oxalicum* 2-HL-M-6	[91]
C_16_H_15_O_9_/*351*	Auxarthrol C 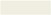	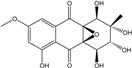	(1*S*,2*R*,3*R*,4*R*,4a*R*,9a*S*)-1,2,3,4,5-pentahydroxy-7-methoxy-2-methyl-1,2,3,4-tetrahydro-4a,9a-epoxyanthraquinone	*Stemphylium* sp. 33231	[93]
C_19_H_11_O_7_/*351*	8-*O*-MethylversicolorinB 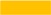	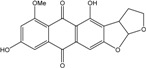	4,8-dihydroxy-6-methoxy-3,3a-dihydroanthra[2,3-b]furo[3,2-d]furan-5,10(2*H*,12a*H*)-dione	*A. versicolor endolichenic*	[108]
C_21_H_20_O_5_/*352*	6,8-Dimethoxy-1-methyl-2-(3-oxobutyl)anthraquinone	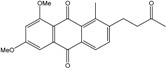	----	*Fusarium* sp. ZZF60	[67]
C_19_H_13_O_7_/*353*	8-*O*-Methylversicolorin A 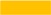	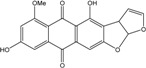	4,8-dihydroxy-6-methoxyanthra[2,3-b]furo[3,2-d]furan-5,10(3a*H*,12a*H*)-dione	*A. versicolor endolichenic*	[108]
C_20_H_18_O_6_/*354*	Averythrin 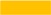	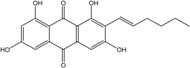	(*E*)-2-(hex-1-en-1-yl)-1,3,6,8-tetrahydroxyanthraquinone	*A.* sp. SCSIO F063 *A. versicolor endolichenic*	[55] [108]
C_30_H_18_O_10_/*358*	Skyrin 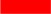	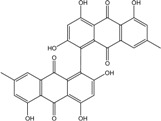	2,2′,4,4′,5,5′-hexahydroxy-7,7′-dimethyl-[1,1′-bianthracene]-9,9′,10,10′-tetraone	*P. chrysogenum*	[89]
C_16_H_12_O_8_S/*364*	Macrosporin-7-*O*-sulfate 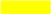	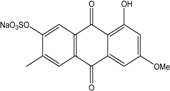	Sodium 8-hydroxy-6-methoxy-3-methyl-9,10-dioxo-9,10-dihydroanthracen-2-yl sulfate	*Stemphylium* sp. *33231*	[93]
C_15_H_9_O_9_S/*365*	Citreorosein-3-*O*-sulfate 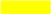	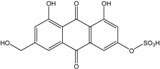	4,5-dihydroxy-7-(hydroxymethyl)-9,10-dioxo-9,10-dihydroanthracen-2-yl hydrogen sulfate	*P. oxalicum* 2-HL-M-6	[91]
C_20_H_14_O_7_/*365*	6,8-di-*O*-methylversico-lorinA	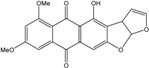	4-hydroxy-6,8-dimethoxyanthra[2,3-b]furo[3,2-d]furan-5,10(3a*H*,12a*H*)-dione	*A. versicolor endolichenic A. versicolor* EN-7 (Genbank no EU042148)	[108] [58]
C_21_H_19_O_6_/*367*	8-*O*-Methylaverythrin 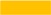	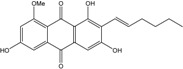	(*E*)-2-(hex-1-en-1-yl)-1,3,6-trihydroxy-8-methoxyanthraquinone	*A. versicolor endolichenic*	[108]
C_20_H_16_O_7_/*368*	Aversin	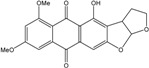	4-hydroxy-6,8-dimethoxy-3,3a-dihydroanthra[2,3-b]furo[3,2-d]furan-5,10(2*H*,12a*H*)-dione	*A. versicolor endolichenic*	[108]
C_20_H_16_O_7_/*368*	Aversin : (−)-isomer	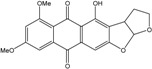	4-hydroxy-6,8-dimethoxy-3,3a-dihydroanthra[2,3-b]furo[3,2-d]furan-5,10(2*H*,12a*H*)-dione	*A. versicolor* EN-7 (Genbank no EU042148)	[58]
C_20_H_20_O_7_/*372*	Averantin	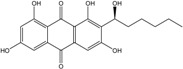	(*S*)-1,3,6,8-tetrahydroxy-2-(1-hydroxyhexyl)anthraquinone	*A.* sp. SCSIO F063	[55]
C_20_H_20_O_7_/*372*	Averantin = (*S*)-(−)-averantin	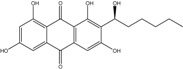	(*S*)-1,3,6,8-tetrahydroxy-2-(1-hydroxyhexyl)anthraquinone	*A.* sp. SCSIO F063	[55]
C_21_H_18_O_7_/*382*	6-*O*-Methylaverufin	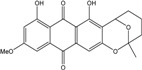	7,9-dihydroxy-11-methoxy-2-methyl-3,4,5,6-tetrahydro-2*H*-2,6-epoxyanthra[2,3-b]oxocine-8,13-dione	Fungus ZSUH-36 *A. versicolor* EN-7	[65] [58]
C_20_H_16_O_8_/*384*	Nidurufin	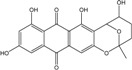	5,7,9,11-tetrahydroxy-2-methyl-3,4,5,6-tetrahydro-2*H*-2,6-epoxyanthra[2,3-b]oxocine-8,13-dione	Fungus Isolate 1850 and isolate 2526	[64]
C_20_H_15_O_8_/*386*	Averufin	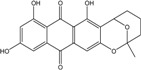	7,9,11-trihydroxy-2-methyl-3,4,5,6-tetrahydro-2*H*-2,6-epoxyanthra[2,3-b]oxocine-8,13-dione	*A. versicolor* Fungus ZSUH-36 Fungus Isolate 1850 and isolate 2526	[109] [65] [64]
C_21_H_22_O_7_/*386*	1′-*O*-Methylaverantin 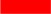	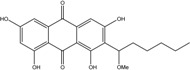	1,3,6,8-tetrahydroxy-2-(1-methoxyhexyl)anthraquinone	*A.* sp. SCSIO F063 *Fungus* ZSUH-36	[55] [65]
C_19_H_15_O_9_/*388*	(2*S*)-2,3-Dihydroxy-propyl-1,6,8-trihydroxy-3-methyl-9,10-dioxoanthracene-2-carboxylate	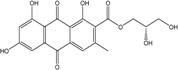	(1*S*,5′*S*,6′*R*,7′*S*,8′*R*)-1′,2,5′,6′,7′,8,8′-heptahydroxy-3′,6-dimethoxy-3,6′-dimethyl-5′,6′,7′,8′,8′a,10′a-hexahydro-[1,2′-bianthracene]-9,9′,10,10′-tetraone	*A. variecolor* B-17	[56]
C_20_H_17_ClO_6_/*388*	7-Chloroaverythrin	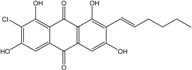	(*E*)-2-chloro-7-(hex-1-en-1-yl)-1,3,6,8-tetrahydroxyanthraquinone	*A.* sp. SCSIO F063	[55]
C_20_H_20_O_8_/*388*	6,8-Di-*O*-methylversiconol	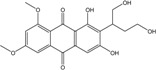	2-(1,4-dihydroxybutan-2-yl)-1,3-dihydroxy-6,8-dimethoxyanthraquinone	*A. versicolor* EN-7 (Genbank no EU042148)	[58]
C_22_H_20_O_7_/*396*	6,8-Di-*O*-methylaverufin 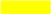	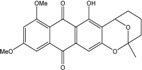	7-hydroxy-9,11-dimetho-xy-2-methyl-3,4,5,6-tetra-hydro-2*H*-2,6-epoxyanthra[2,3-b]oxocine-8,13-dione	*A. versicolor endolichenic*	[108]
C_22_H_20_O_7_/*396*	6,8-Di-*O*-methylnidurufin	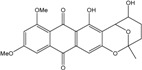	5,7-dihydroxy-9,11-dime-thoxy-2-methyl-3,4,5,6-tetrahydro-2*H*-2,6-epoxyanthra[2,3-b]oxocine-8,13-dione	*A. versicolor endolichenic A. versicolor* EN-7 (Genbank no EU042148)	[108] [58]
C_22_H_22_O_7_/*398*	6,8-Di-*O*-methylaverufanin 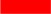	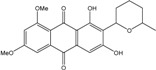	1,3-dihydroxy-6,8-dimethoxy-2-(6-methyltetra-hydro-2*H*-pyran-2-yl)anthracene-9,10-dione	*Fungus* ZSUH-36	[65]
C_22_H_24_O_7_/*400*	6,1′-*O*,*O*-Dimethylaverantin 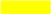	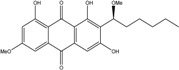	(*S*)-1,3,8-trihydroxy-6-methoxy-2-(1-methoxyhexyl)anthraquinone	*A.* sp. SCSIO F063	[55]
C_20_H_17_O_9_/*401*	Variecolorquinone A 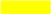	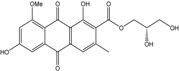	(*S*)-2,3-dihydroxypropyl 1,6-dihydroxy-8-methoxy-3-methyl-9,10-dioxo-9,10-dihydroanthracene-2-carboxylate	*A.glaucus A. variecolor* B-17	[50,51,52,53] [56]
C_22_H_24_O_7_/*401*	6,8-Di-*O*-methylaveran-tin 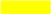	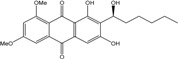	(*S*)-1,3-dihydroxy-2-(1-hydroxyhexyl)-6,8-dimetho-xyanthraquinone	*A. versicolor* EN-7 (Genbank no EU042148) *A.* sp. SCSIO F063	[58] [55]
C_21_H_19_ClO_6_/*402*	6-*O*-Methyl-7-chloroaverythrin 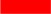	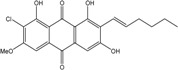	(*E*)-2-chloro-7-(hex-1-en-1-yl)-1,6,8-trihydroxy-3-methoxyanthraquinone	*A.* sp. SCSIO F063	[55]
C_20_H_19_ClO_7_/*407*	(1′S)-7-Chloroaverantin 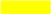	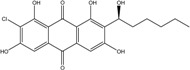	(*S*)-2-chloro-1,3,6,8-tetrahydroxy-7-(1-hydroxyhexyl)anthraquinone	*A.* sp. SCSIO F063	[55]
C_22_H_23_ClO_7_/*407*	6,1′-*O*,*O*-Dimethyl-7-chloroaverantin 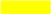	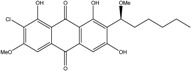	(*S*)-2-chloro-1,6,8-trihydroxy-3-methoxy-7-(1-methoxyhexyl)anthraquinone	*A.* sp. *SCSIO F063*	[55]
C_23_H_26_O_7_/*414*	6,8,1′-Tri-*O*-methyl-averantin 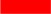	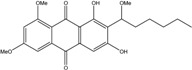	1,3-dihydroxy-6,8-dimethoxy-2-(1-methoxyhexyl)anthraquinone	*A.versicolor endolichenic* Fungus ZSUH-36	[108] [65]
C_21_H_19_O_9_/*415*	6-3-*O*-(Ribofuranosyl)questin	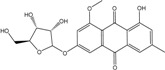	1,6-dihydroxy-6-*O*-(ribofuranosyl)-8-methoxy-3-methylanthraquinone	*Eurotium rubrum*	[63]
C_21_H_21_ClO_7_/*420*	1′-*O*-methyl-7-chloro averantin 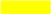	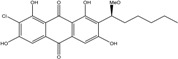	(*S*)-2-chloro-1,3,6,8-tetrahydroxy-7-(1-methoxy-hexyl)anthraquinone	*A.* sp. SCSIO F063	[55]
C_21_H_21_ClO_7_/*421*	6-*O*-methyl-7-chloro-averantin 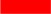	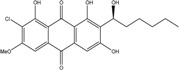	(*S*)-2-chloro-1,6,8-trihydroxy-7-(1-hydroxyhexyl)-3-methoxyanthraquinone	*A.* sp. SCSIO F063	[55]
C_24_H_28_O_8_/*444*	Averantin-1′-butyl ether	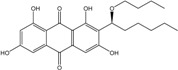	(*S*)-2-(1-butoxyhexyl)-1,3,6,8-tetrahydroxy-anthraquinone	*A.* sp. SCSIO F063	[55]
C_24_H_27_ClO_7_/*463*	7-Chloroaverantin-1′-butyl ether 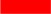	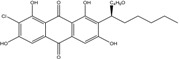	(*S*)-2-(1-butoxyhexyl)-7-chloro-1,3,6,8-tetrahy-droxyanthraquinone	*A.* sp. SCSIO F063	[55]
C_21_H_21_BrO_7_/*465*	6-*O*-Methyl-7-bromoaverantin 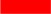	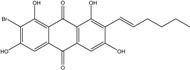	(*S*)-2-bromo-1,6,8-trihydroxy-7-(1-hydroxyhexyl)-3-methoxyanthraquinone	*A.* sp. SCSIO F063	[55]
C_22_H_23_BrO_7_/*479*	6,1′-*O*,*O*-Dimethyl-7-bromoaverantin 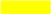	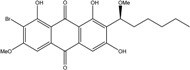	(*S*)-2-bromo-1,6,8-trihydroxy-3-methoxy-7-(1-methoxyhexyl)anthraquinone	*A.* sp. SCSIO F063	[55]
C_24_H_23_O_11_/*487*	Macrosporin2-*O*-(6′-acetyl)-a-d-glucopyranoside 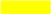	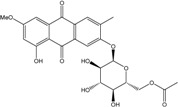	((2*R*,3*S*,4*S*,5*R*,6*R*)-3,4,5-trihydroxy-6-((8-hydroxy-6-methoxy-3-methyl-9,10-dioxo-9,10-dihydroanthracen-2-yl)oxy)tetrahydro-2H-pyran-2-yl)methyl acetate	*Stemphylium* sp. *33231*	[93]
C_28_H_24_O_10_/*520*	Penicillanthranin A 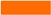	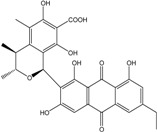	(1*S*,3*R*,4*S*)-1-(6-ethyl-1,3,8-trihydroxy-9,10-dioxo-9,10-dihydroanthracen-2-yl)-6,8-dihy-droxy-3,4,5-trimethyliso-chroman-7-carboxylic acid	*P. citrinum* PSU-F51	[88]
C_28_H_24_O_11_/*536*	Penicillanthranin B 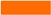	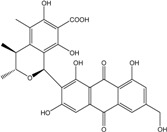	(1*S*,3*R*,4*S*)-6,8-dihydroxy-3,4,5-trimethyl-1-(1,3,8-trihydroxy-6-(hydroxy-methyl)-9,10-dioxo-9,10-dihydroanthracen-2-yl)isochroman-7-carboxylic acid	*P. citrinum* PSU-F51	[88]
C_32_H_24_O_8_/*536*	(*trans*)-*R* (*cis*)-Emodin-Physcion bianthrone	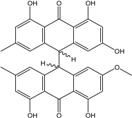	2,4,4′,5,5′-pentahydroxy-2′-methoxy-7,7′-dimethyl-[9,9′-bianthracene]-10,10′(9*H*,9′*H*)-dione	*A. glaucus*	[50,51,52,53]
C_32_H_21_O_10_/*565*	Alterporriol Q 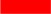	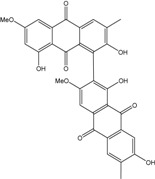	1′,2,7′,8-tetrahydroxy-3′,6-dimethoxy-3,6′-dimethyl-[1,2′-bianthracene]-9,9′,10,10′-tetraone	*Al.* sp. ZJ-2008003	[48]
C_32_H_21_O_10_/*565*	Alterporriol R 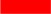	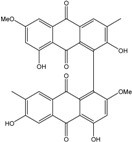	2,4′,6′,8-tetrahydroxy-2′,6-dimethoxy-3,7′-dimethyl-[1,1′-bianthracene]-9,9′,10,10′-tetraone	*Al.* sp. ZJ-2008003	[48]
C_32_H_22_O_10_/*566*	Alterporriol V 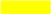	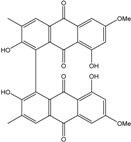	2,2′,8,8′-tetrahydroxy-6,6′-dimethoxy-3,3′-dimethyl-[1,1′-bianthracene]-9,9′,10,10′-tetraone	*Stemphylium* sp. 33231	[93]
C_30_H_22_O_12_/*574*	Cytoskyrin A	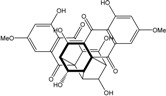	(6*R*,14*R*,17*S*,18*R*,19*R*,20*S*)-1,7,9,15,17,20-hexahydroxy-3,11-dimethoxy-6,13a,5a,14-(epibutane[1,2,3,4]tetrayl)cycloocta[1,2-b:5,6-b′]dinaphtha-lene-5,8,13,16(6*H*,14*H*)-tetraone	*Curvularia lunata*	[50,51,59,60]
C_32_H_26_O_10_/*586*	Alterporriol K 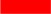	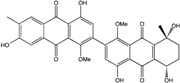	(5*S*,8*R*)-4,4′,5,7′,8-penta-hydroxy-1,1′-dimethoxy-6′,8-dimethyl-5,6,7,8-tetrahydro-[2,2′-bianthracene]-9,9′,10,10′-tetraone	*Al.* sp. ZJ9-6B *Al.* sp. ZJ-2008003	[49] [48]
C_32_H_30_O_13_/*590*	Alterporriol T 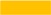	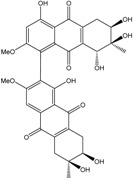	(6*R*,6′*S*,7*R*,7′*R*,8*R*)-1′,4,6,6′,7,7′,8-heptahydroxy-2,3′-dimethoxy-6′,7-dime-thyl-5,5′,6,6′,7,7′,8,8′-octahydro-[1,2′-bianthracene]-9,9′,10,10′-tetraone	*Stemphylium* sp. 33231	[93]
C_32_H_25_O_12_/*601*	Alterporriol L 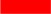	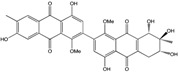	(6*S*,7*R*,8*R*)-4,4′,6,7,7′,8-hexahydroxy-1,1′-dimethoxy-6′,7-dimethyl-5,6,7,8-tetrahydro-[2,2′-bianthracene]-9,9′,10,10′-tetraone	*Al.* sp. ZJ9-6B *Al.* sp. ZJ-2008003	[49] [48]
C_32_H_25_O_12_/*601*	Alterporriol M 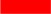	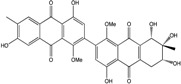	(6*S*,7*S*,8*R*)-4,4′,6,7,7′,8-hexahydroxy-1,1′-dimethoxy-6′,7-dimethyl-5,6,7,8-tetrahydro-[2,2′-bianthracene]-9,9′,10,10′-tetraone	*Al.* sp. ZJ9-6B *Al.* sp. ZJ-2008003	[49] [48]
C_32_H_25_O_12_/*601*	Alterporriol P 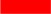	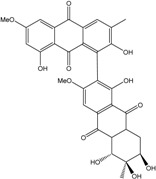	(5′*R*,6′*R*,7′*R*)-1′,2,5′,6′,7′,8-hexahydroxy-3′,6-dimethoxy-3,6′-dimethyl-5′,6′,7′,8′,8′a,10′a-hexahydro-[1,2′-bianthracene]-9,9′,10,10′-tetraone	*Al.* sp. ZJ-2008003	[48]
C_32_H_26_O_12_/*602*	Alterporriol W 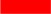	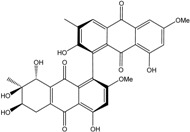	(1′*R*,6*R*,7*R*,8*R*)-2′,4,6,7,8,8′-hexahydroxy-2,6′-dimethoxy-3′,7-dimethyl-5,6,7,8-tetrahydro-[1,1′-bianthracene]-9,9′,10,10′-tetraone	*Stemphylium* sp. 33231	[93]
C_32_H_30_O_12_/*606*	Alterporriol U 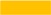	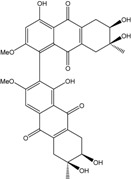	(6*R*,6′*S*,7*S*,7′*R*)-1′,4,6,6′,7,7′-hexahydroxy-2,3′-di-methoxy-6′,7-dimethyl-5,5′,6,6′,7,7′,8,8′-octahy-dro-[1,2′-bianthracene]-9,9′,10,10′-tetraone	*Stemphylium* sp. 33231	[93]
C_31_H_32_O_13_/*612*	Alterporriol S 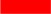	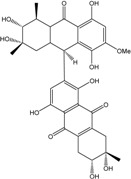	(2′*S*,3′*R*,4′*S*,6*R*,7*S*,9′*R*)-1,2′,3′,4,5′,6,7,8′-octahy-droxy-7′-methoxy-2′,4′,7-trimethyl-2′,3′,4′,4′a,5,6,7,8,9′,9′a-decahydro-[2,9′-bianthracene]-9,10,10′(1′*H*)-trione	*Al.* sp. (SK11)	[47]
C_32_H_25_O_13_/*617*	(+)-aS-alterporriol C 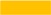	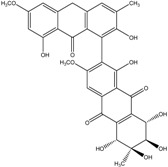	(1*S*,5′*S*,6′*R*,7′*S*,8′*R*)-1′,2,5′,6′,7′,8,8′-heptahydroxy-3′,6-dimethoxy-3,6′-dime-thyl-5′,6′,7′,8′,8′a,10′a-hexahydro-[1,2′-bianthracene]-9,9′,10,10′-tetraone	*Al.* sp. (SK11)	[47]
C_32_H_25_O_13_/*617*	Alterporriol C 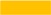	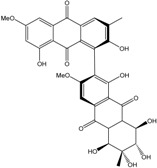	(1*S*,5′*R*,6′*S*,7′*R*,8′*S*)-1′,2,5′,6′,7′,8,8′-heptahydroxy-3′,6-dimethoxy-3,6′-dime-thyl-5′,6′,7′,8′,8′a,10′a-hexahydro-[1,2′-bianthracene]-9,9′,10,10′-tetraone	*Al.* sp. (SK11) *Al.* sp. ZJ-2008003	[47] [48]
C_32_H2_9_O_14_/*637*	Alterporriol N 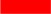	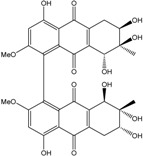	(6*R*,6′*R*,7*R*,7′*R*,8*R*,8′*R*)-4,4′,6,6′,7,7′,8,8′-octahy-droxy-2,2′-dimethoxy-7,7′-dimethyl-5,5′,6,6′,7,7′,8,8′-octahydro-[1,1′-bianthracene]-9,9′,10,10′-tetraone	*Al.* sp. ZJ-2008003	[48]
C_32_H2_9_O_14_/*637*	Alterporriol O 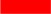	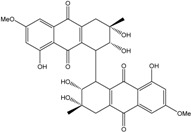	(2*R*,2′*R*,3*S*,3′*S*)-2,2′,3,3′,8,8′-hexahydroxy-6,6′-dimethoxy-3,3′-dimethyl-1,1′,2,2′,3,3′,4,4′-octahy-dro-[1,1′-bianthracene]-9,9′,10,10′-tetraone	*Al.* sp. ZJ-2008003	[48]
C_34_H_33_O_17_/*713*	Acetylalterporriol D 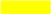	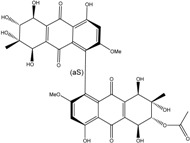	(1′*S*,5*S*,5′*S*,6*R*,6′*R*,7*S*,7′*S*,8*R*,8′*R*)-4,4′,5,5′,6′,7,7′,8,8′-nonahydroxy-2,2′-dimethoxy-7,7′-dimethyl-9,9′,10,10′-tetraoxo-5,5′,6,6′,7,7′,8,8′,9,9′,10,10′-dodecahydro-[1,1′-bianthracen]-6-yl acetate	*Stemphylium globuliferum*	[94]
C_34_H_33_O_17_/*713*	Acetylalterporriol E 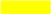	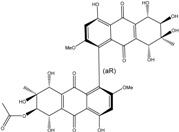	(1′*R*,5*S*,5′*S*,6*R*,6′*R*,7*S*,7′*S*,8*R*,8′*R*)-4,4′,5,5′,6′,7,7′,8,8′-nonahydroxy-2,2′-dimethoxy-7,7′-dimethyl-9,9′,10,10′-tetraoxo-5,5′,6,6′,7,7′,8,8′,9,9′,10,10′-dodecahydro-[1,1′-bianthracen]-6-yl acetate	*Stemphylium globuliferum*	[94]
C_34_H_33_O_17_/*713*	Alterporriol D 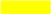	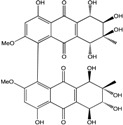	(1*S*,5*S*,5′*S*,6*R*,6′*R*,7*S*,7′*S*,8*R*,8′*R*)-4,4′,5,5′,6,6′,7,7′,8,8′-decahydroxy-2,2′-dimethoxy-7,7′-dimethyl-5,5′,6,6′,7,7′,8,8′-octahy-dro-[1,1′-bianthracene]-9,9′,10,10′-tetraone	*Stemphylium globuliferum*	[94]
C_34_H_33_O_17_/*713*	Alterporriol E 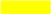	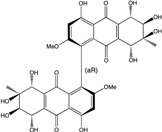	(1*R*,5*S*,5′S,6*R*,6′*R*,7*S*,7′*S*,8*R*,8′*R*)-4,4′,5,5′,6,6′,7,7′,8,8′-decahydroxy-2,2′-dimethoxy-7,7′-dimethyl-5,5′,6,6′,7,7′,8,8′-octahy-dro-[1,1′-bianthracene]-9,9′,10,10′-tetraone	*Stemphylium globuliferum*	[94]
C_48_H_40_O_21_/*952*	Stemphylanthranol A	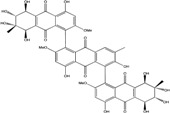	(5*S*,5′′*S*,6*R*,6′′*R*,7*S*,7′′*S*,8*R*,8′′*R*)-2′,4,4′′,5,5′′,6,6′′,7,7′′,8,8′,8′′-dodecahydroxy-2,2′′,6′-trimethoxy-3′,7,7′′-trimethyl-5,5′′,6,6′′,7,7′′,8,8′′-octahydro-[1,1′:5′,1′′-teranthracene]-9,9′,9′′,10,10′,10′′-hexaone	*Stemphylium globuliferum*	[94]
C_48_H_40_O_21_/*952*	Stemphylanthranol B	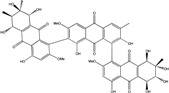	(5*S*,5′′*R*,6*R*,6′′*R*,7S,7′′*R*,8*R*,8′′*R*)-2′,4,4′′,5,5′′,6,7,7′′,8,8′,8′′-undecahydroxy-2,2′′,6′-trimethoxy-3′,6′′,7,7′′-tetramethyl-5,5′′,6,6′′,7,7′′,8,8′′-octahydro-[1,1′:7′,1′′-tetranthracene]-9,9′,9′′,10,10′,10′′-hexaone	*Stemphylium globuliferum*	[94]
----	7-Chloroemodic acid	----	----	*Caloplaca* spp. *(*e.g., *C. ehrenbergii, C. schaereri, C. spitsbergensis, etc.)*	[75,76,77,79,96]
----	7-Chloroemodinal	----	----	*Caloplaca* spp. *(*e.g., *C. ehrenbergii, C. schaereri, C. spitsbergensis, etc.)L. hafellneri L. leprolytoides*	[75,76,77,79,96] [80]
----	7-Chloro-1,6,8-trihydroxy-3-methyl-10-anthrone	----	----	*Caloplaca* spp. *(*e.g., *C. ehrenbergii, C. schaereri, C. spitsbergensis, etc.)*	[75,76,77,79,96]

Abbreviations: *A.*: *Aspergillus*; *Al*.: *Alternaria*; *P*.: *Penicillium*. Orange brown: 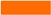; Orange: 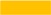; Yellow: 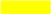; Red: 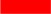; Bronze: 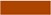.

**Table 3:** Effective anthraquinones with a protein kinase CK2 inhibitor activity (adapted from Meggio *et al.* [218]).

Name	Structure	IC_50_ (µM)—K*_i_* (µM)
Emodin (3-methyl-1,6,8-trihydroxyanthraquinone)	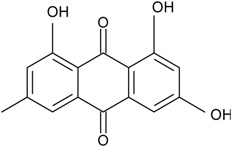	K*_i_* = 1.85
1,8-dihydroxy-4-nitro-anthracene-9,10-dione	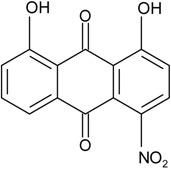	IC_50_ = 0.30; K*_i_* = 0.78
1,8-dihydroxy-3-methyl-4-nitro-anthracene-9,10-dione	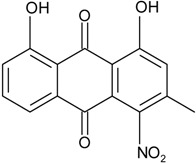	IC_50_ = 0.30; K*_i_* = 0.95
1,8-dihydroxy-4,5-dinitro-anthracene-9,10-dione	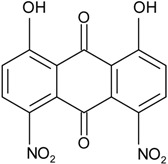	IC_50_ = 0.30
1,4-dihydroxy-5,8-diamino-anthracene-9,10-dione	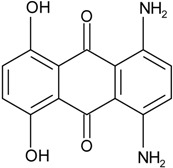	IC_50_ = 0.30; K*_i_* = 0.42
1-bromo-4,5-dihydroxy-8-nitro-anthracene-9,10-dione	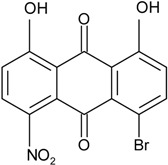	IC_50_ = 0.40
1,4,5-trihydroxy-8-(2-bromoacetamido)-anthracene-9,10-dione	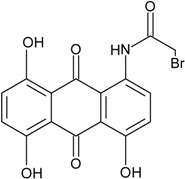	IC_50_ = 0.70
